# Supplementary material for: Oxygen-tolerant CO2 electroreduction over covalent organic frameworks via photoswitching control oxygen passivation strategy
Source: Nat Commun. 2024 Feb 17;15:1479. doi: 10.1038/s41467-024-45959-9 (PMC10874412; doi:10.1038/s41467-024-45959-9)
Supplement: Supplementary file 1 — Supplementary Information [file 41467_2024_45959_MOESM1_ESM.pdf]

Supplementary Information for

Oxygen-tolerant CO_2 electroreduction over covalent organic frameworks via photoswitching control oxygen passivation strategy

Zhu et al.

Outline

1. Supplementary Figures (Figure. 1-53) .... 3-55.

2. Supplementary Tables (Table 1-4) .... 56-61.

3. Supplementary Notes .... 62.

4. Supplementary References ....63.

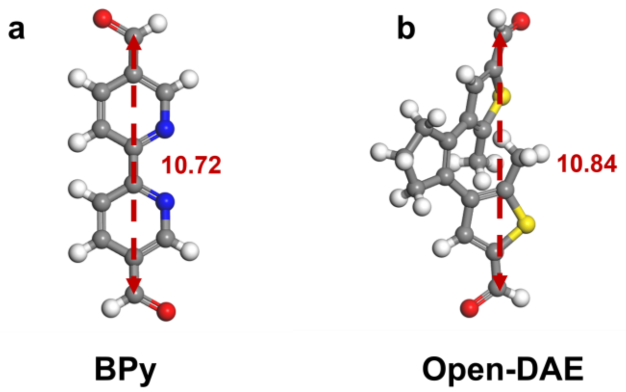

Supplementary Figure 1. The molecule size of DAE **a** and BPy **b**.

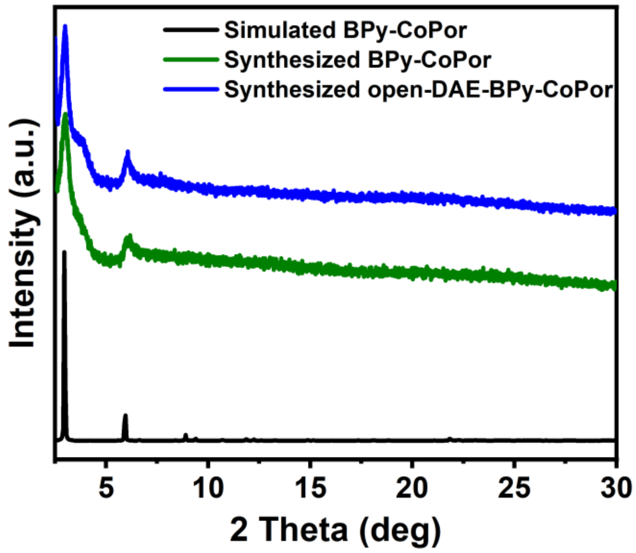

Supplementary Figure 2. The PXRD pattern of simulated and synthesized of BPy-CoPor and open-DAE-BPy-CoPor.

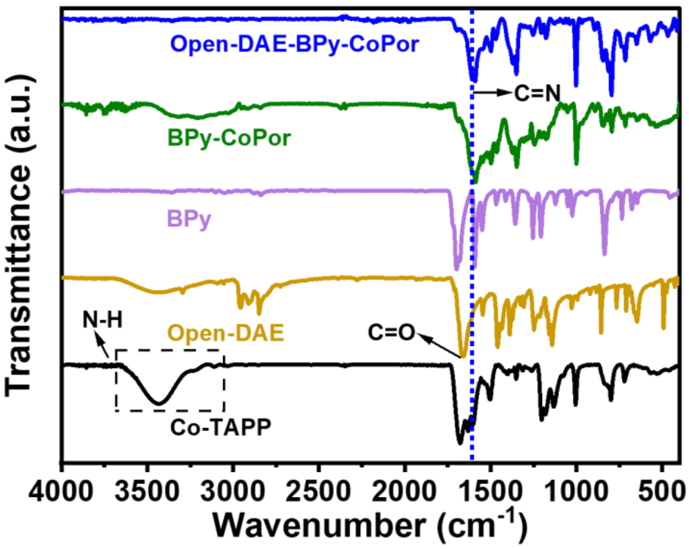

Supplementary Figure 3. The FT-IR of Co-TAPP, open-BPy, DAE, BPy-CoPor and open-DAE-BPy-CoPor.

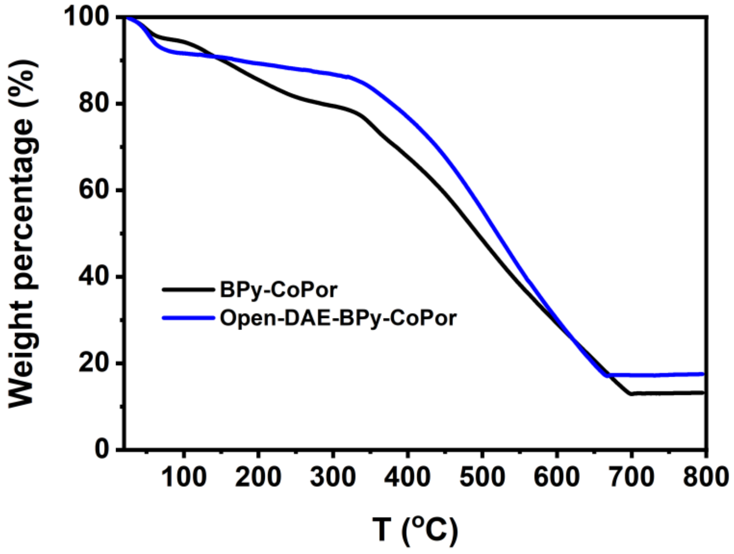

Supplementary Figure 4. TGA spectra of BPy-CoPor and open-DAE-BPy-CoPor.

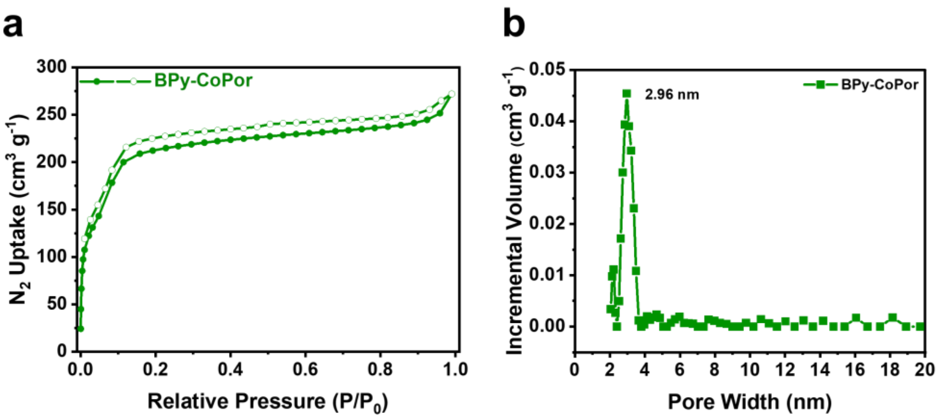

Supplementary Figure 5. The N_2 sorption isotherms **a** and pore width distribution of BPy-CoPor **b**.

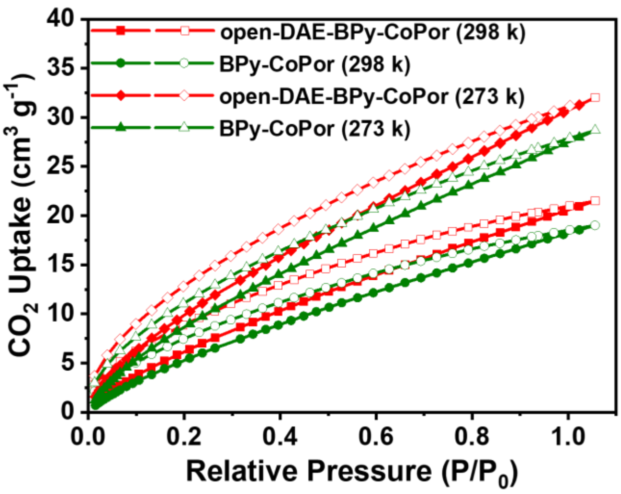

Supplementary Figure 6. The CO_2 sorption isotherms of BPy-CoPor and open-DAE-BPy-CoPor measured at 298 K and 273 K.

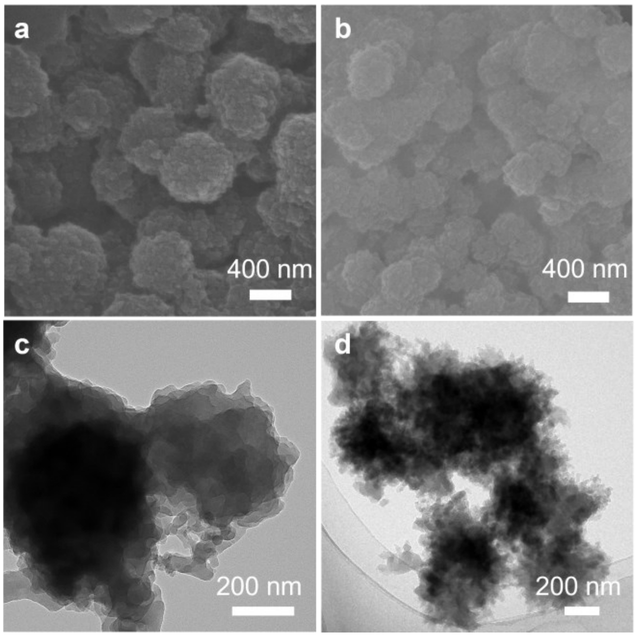

Supplementary Figure 7. The SEM images of **a** BPy-CoPor and **b** open-DAE-BPy-CoPor, and the TEM images of **c** BPy-CoPor and **d** open-DAE-BPy-CoPor.

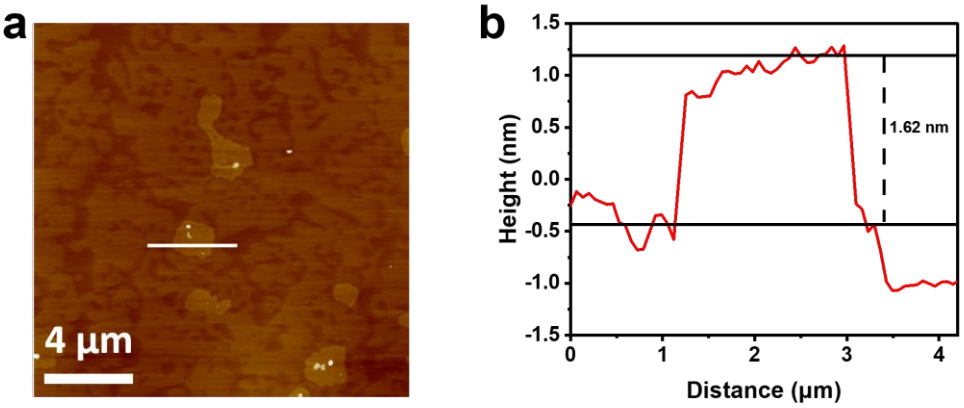

Supplementary Figure 8. The atomic force microscope image (AFM) **a** and the height profile of AFM along the marked white line **b** of open-DAE-BPy-CoPor.

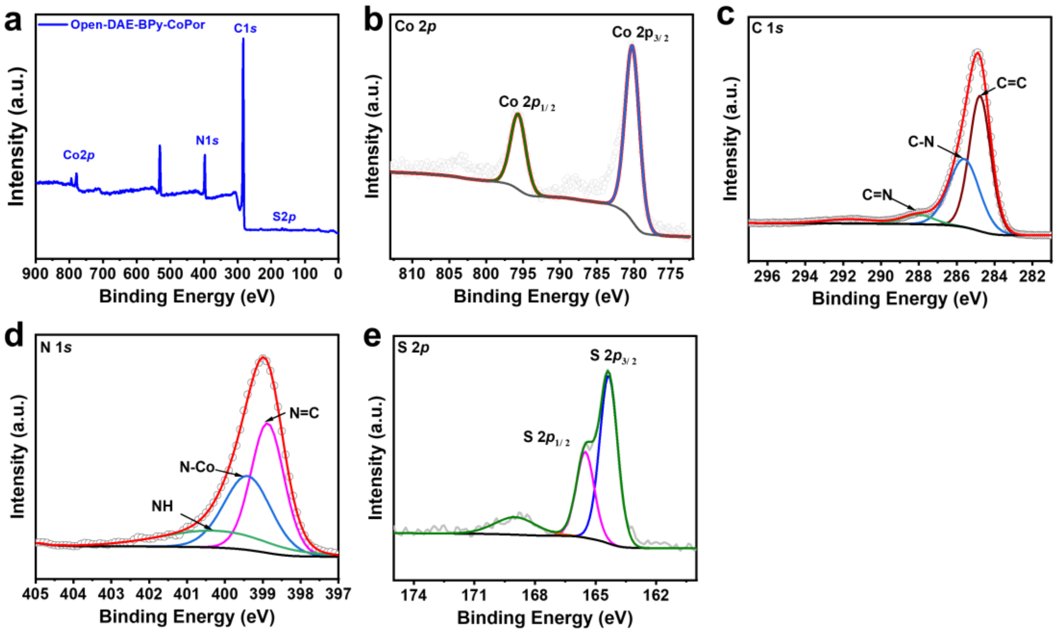

Supplementary Figure 9. High-resolution XPS spectrum of open-DAE-BPy-CoPor. **a** Total XPS spectra, XPS high-resolution scan of **b** Co 2*p*, **c** C 1*s*, **d** N1*s* and **e** S 2*p*.

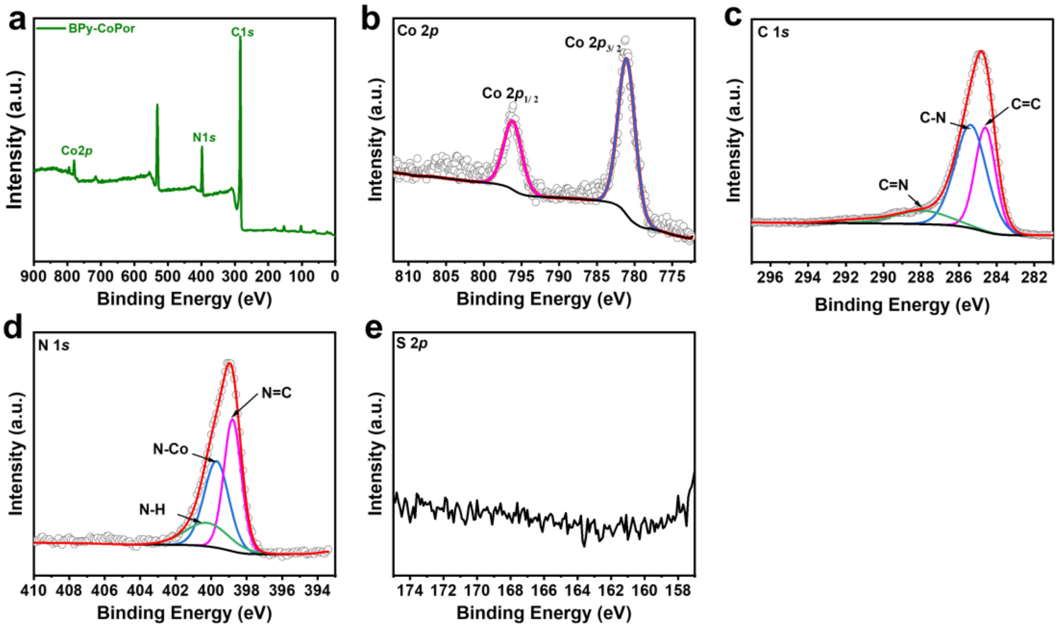

Supplementary Figure 10. High-resolution XPS spectrum of BPy-CoPor. **a** Total XPS spectra, XPS high-resolution scan of **b** Co 2*p*, **c** C 1*s*, **d** N1*s* and **e** S 2*p*.

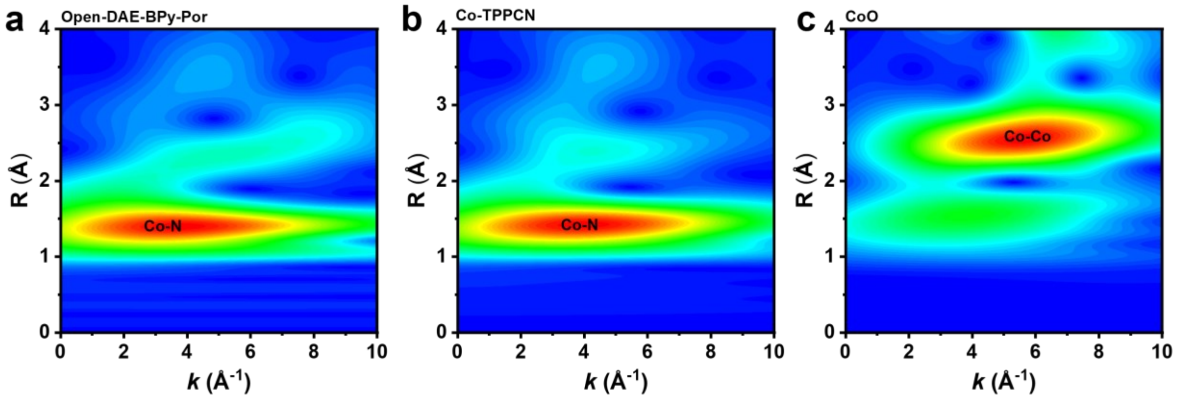

Supplementary Figure 11. Wavelet transformed (WT) EXAFS analysis plots of **a** open-DAE-BPy-CoPor, **b** Co-TPPCN and **c** CoO.

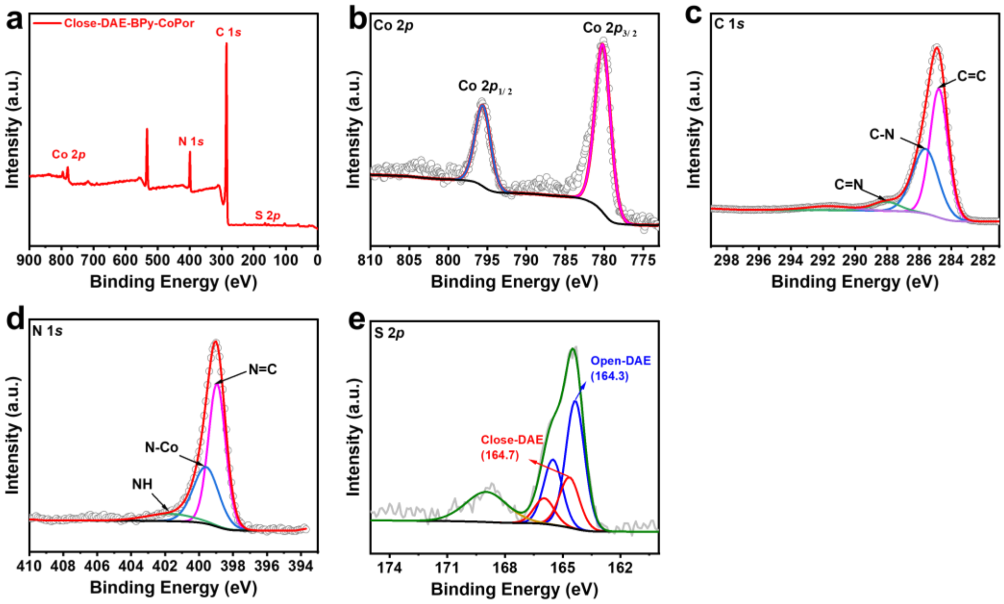

Supplementary Figure 12. High-resolution XPS spectrum of close-DAE-BPy-CoPor. **a** Total XPS spectra, XPS high-resolution scan of **b** Co 2*p*, **c** C 1*s*, **d** N1*s* and **e** S 2*p*.

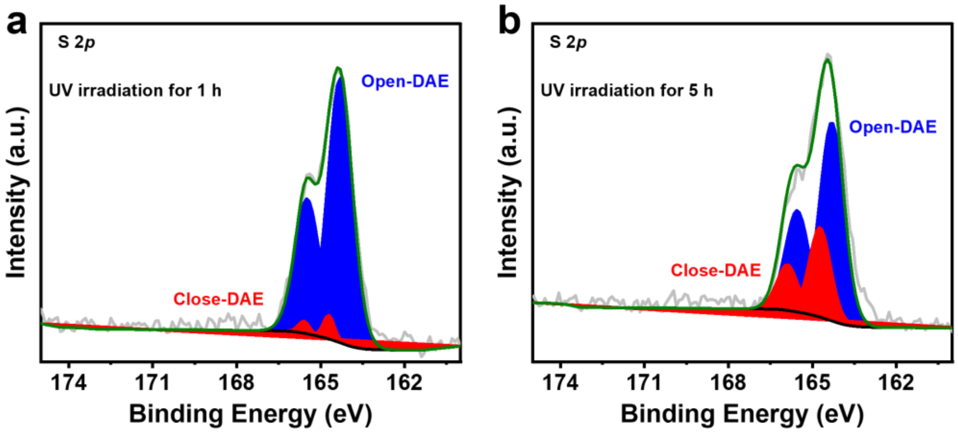

Supplementary Figure 13. The X-ray photoelectron spectroscopy of S 2*p* region for close-DAE-BPy-CoPor following 1 h **a** and 5 h **b** after irradiation by UV light.

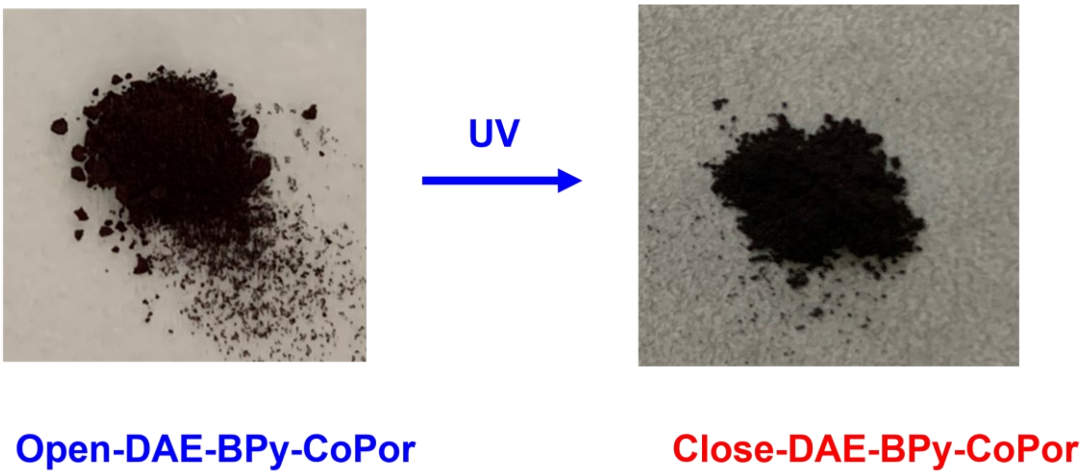

Supplementary Figure 14. The photos of open-DAE-BPy-CoPor and close-DAE-BPy-CoPor.

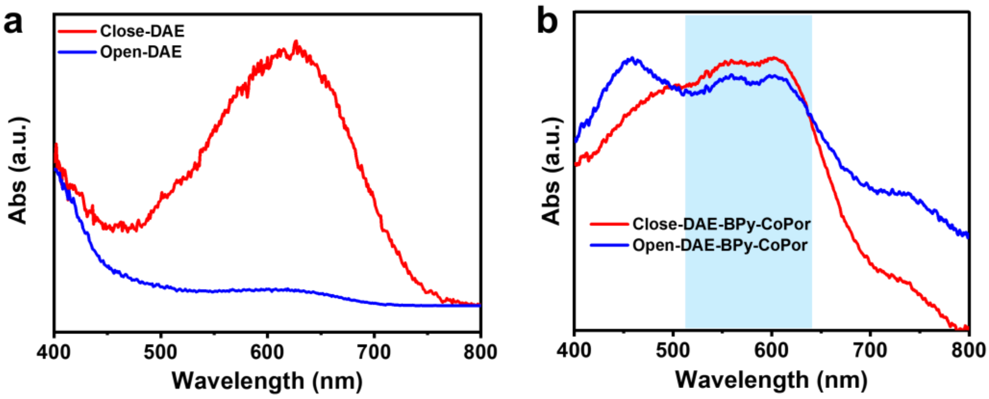

Supplementary Figure 15. The Solid-state UV-vis diffuse reflectance spectra of DAE (open and close form) **a** and open-DAE-BPy-CoPor and close-DAE-BPy-CoPor **b**.

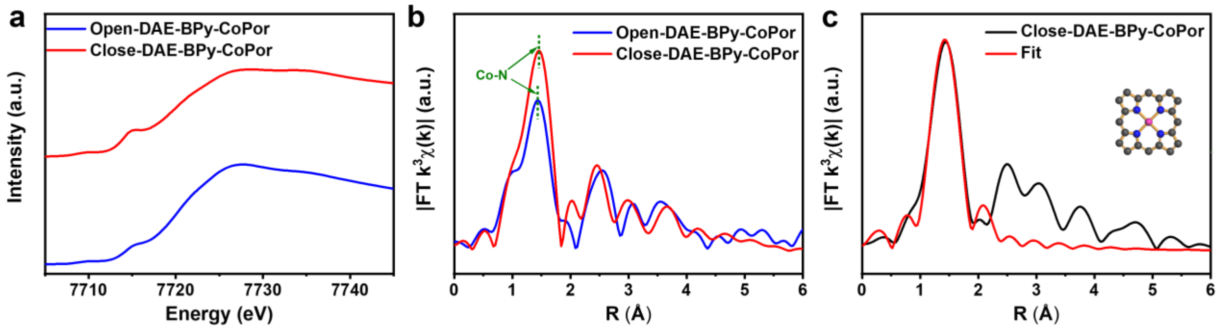

Supplementary Figure 16. The local coordination structure. a Co *K*-edge of X-ray absorption near-edge structure spectra of open-DAE-BPy-CoPor and close-DAE-BPy-CoPor. **b** Co *K*-edge of EXAFS spectra of open-DAE-BPy-CoPor and close-DAE-BPy-CoPor. **c** The extended X-ray absorption fine structure fitting curves of close-DAE-BPy-CoPor.

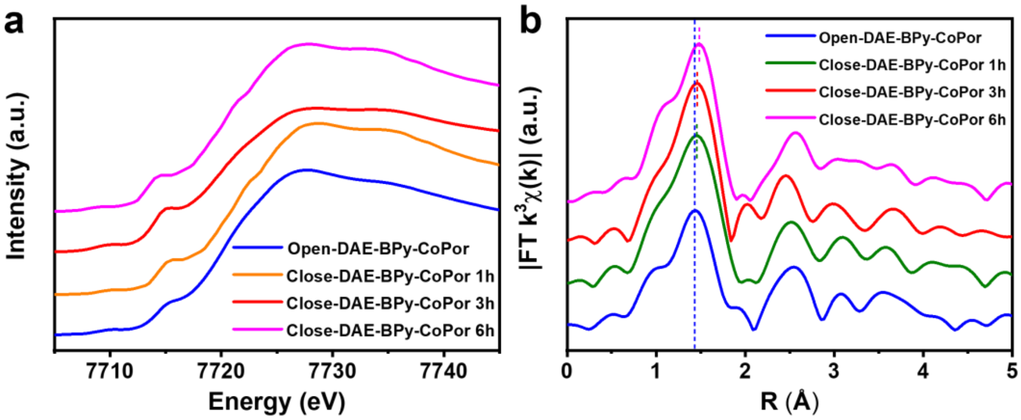

Supplementary Figure 17. The local coordination structure of open-DAE-BPy-CoPor and close-DAE-BPy-CoPor under varying durations of UV irradiation (1 h, 3 h and 6h). a Co *K*-edge of X-ray absorption near-edge structure spectra. **b** Co *K*-edge of EXAFS spectra.

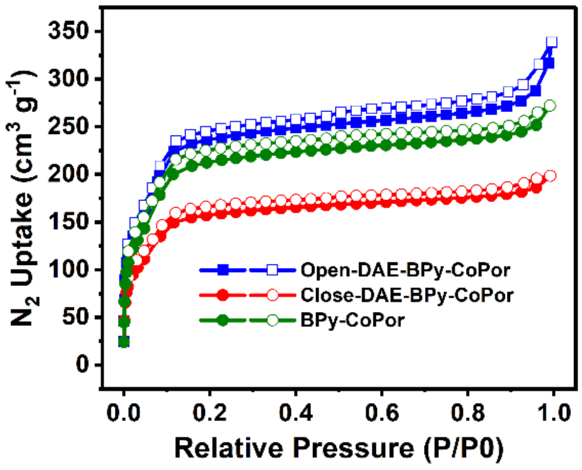

Supplementary Figure 18. The N_2 sorption isotherms of open-DAE-BPy-CoPor, BPy-CoPor and close-DAE-BPy-CoPor.

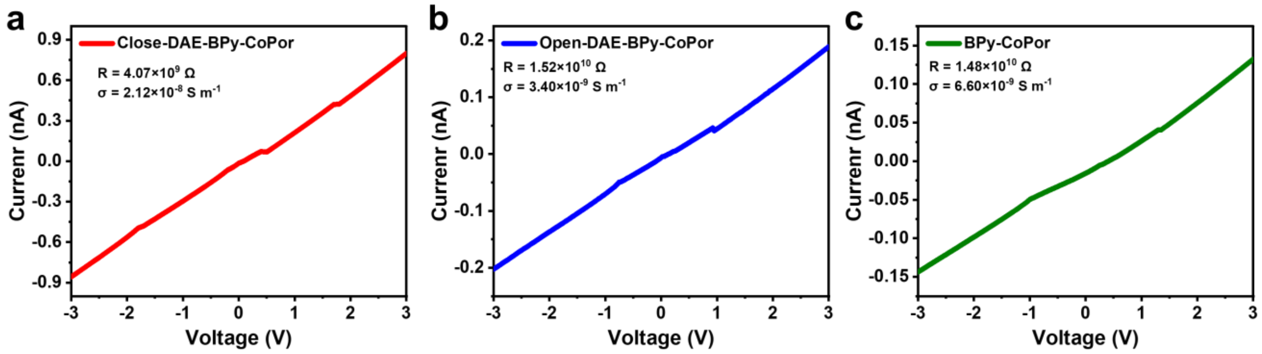

Supplementary Figure 19. Electrical measurement of **a** close-DAE-BPy-CoPor (L = 0.51 mm), **b** open-DAE-BPy-CoPor (L = 0.25 mm) and **c** BPy-CoPor (L = 0.48 mm) were performed using two-electrode in air at a constant temperature of 298 K and absence of light ($\sigma = L/(R\times\pi(d/2)^2)$, d = 2.5 mm).

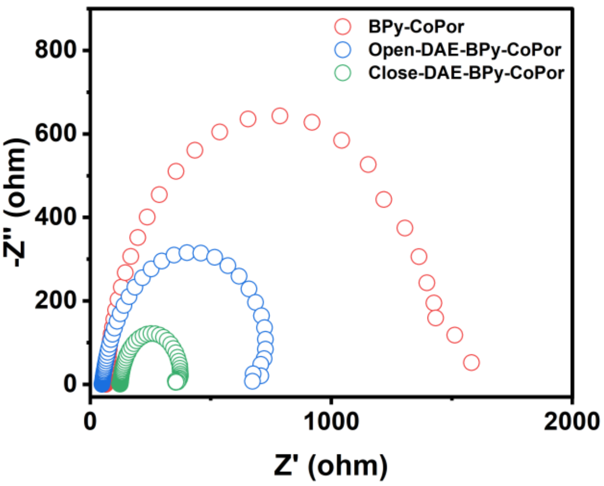

Supplementary Figure 20. Electrochemical impedance spectroscopy (EIS) of open-DAE-BPy-CoPor, close-DAE-BPy-CoPor and BPy-CoPor on FTO at −1.5 V vs Ag/AgCl, with 0.2 M Na_2SO_4 as electrolyte.

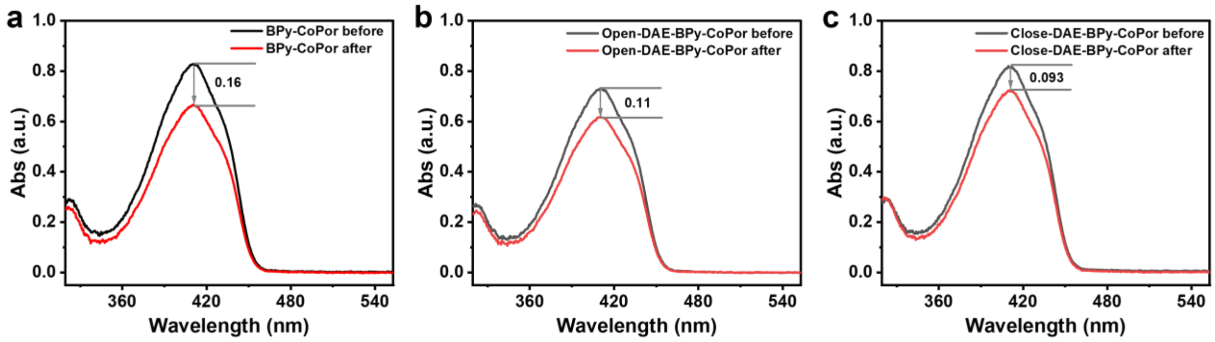

Supplementary Figure 21. The DPBF absorption spectrum of **a** BPy-CoPor, **b** open-DAE-BPy-CoPor and **c** close-DAE-BPy-CoPor in 0.1 M $TBAPF_6$/MeCN at -0.1 V vs. Ag/AgCl.

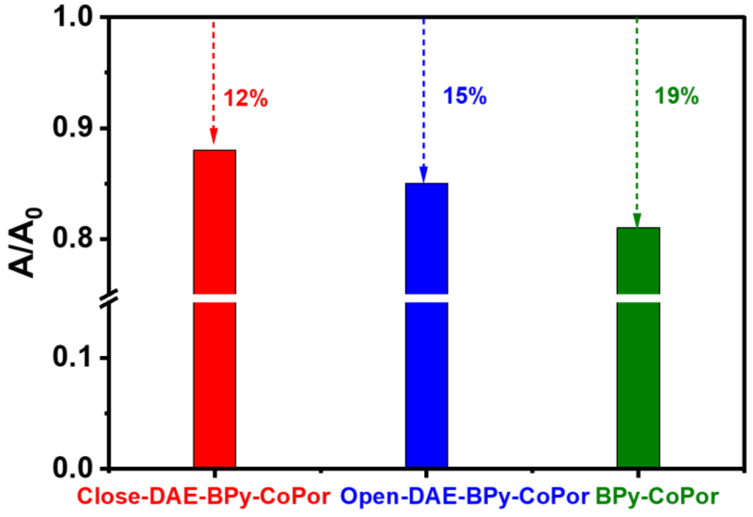

Supplementary Figure 22. The decay rate of DPBF upon close-DAE-BPy-CoPor, open-DAE-BPy-CoPor and BPy-CoPor.

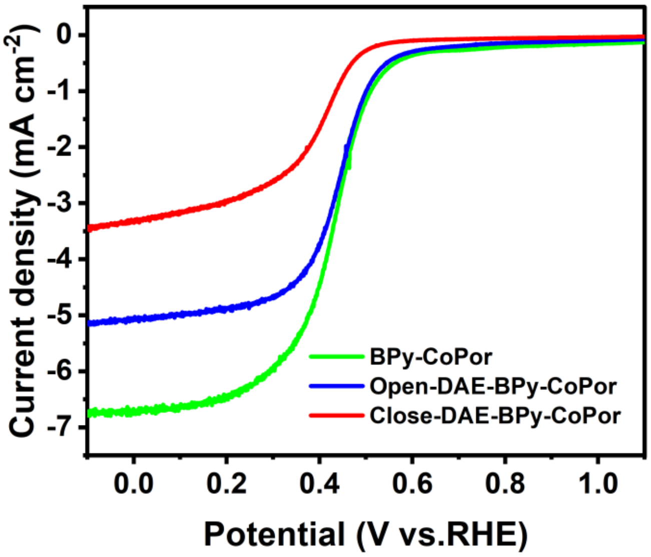

Supplementary Figure 23. Linear sweep voltammograms (LSVs) of BPy-CoPor, open-DAE-BPy-CoPor and close-DAE-BPy-CoPor at 1600 rpm in O_2-saturated 0.2 M Na_2SO_4 (scan rate: 10 mV s^{-1}).

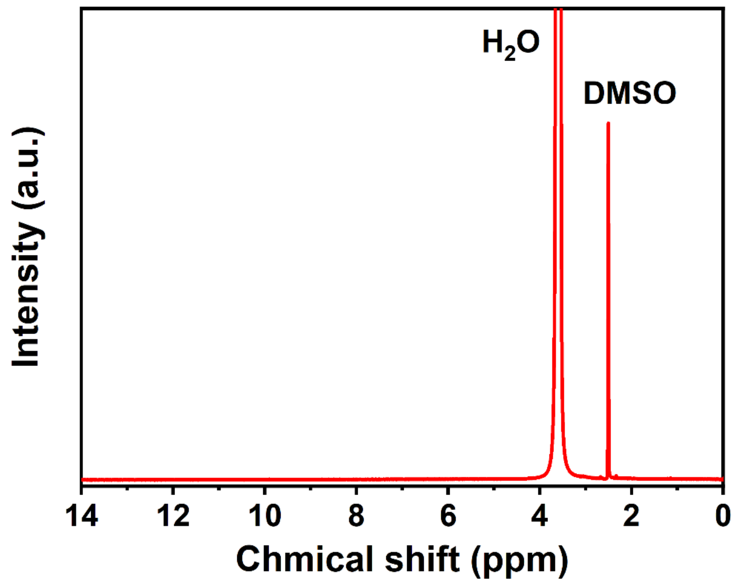

Supplementary Figure 24. ^{1}H NMR characterization of the liquid product during CO_2 reduction process (electrocatalyst, close-DAE-BPy-CoPor).

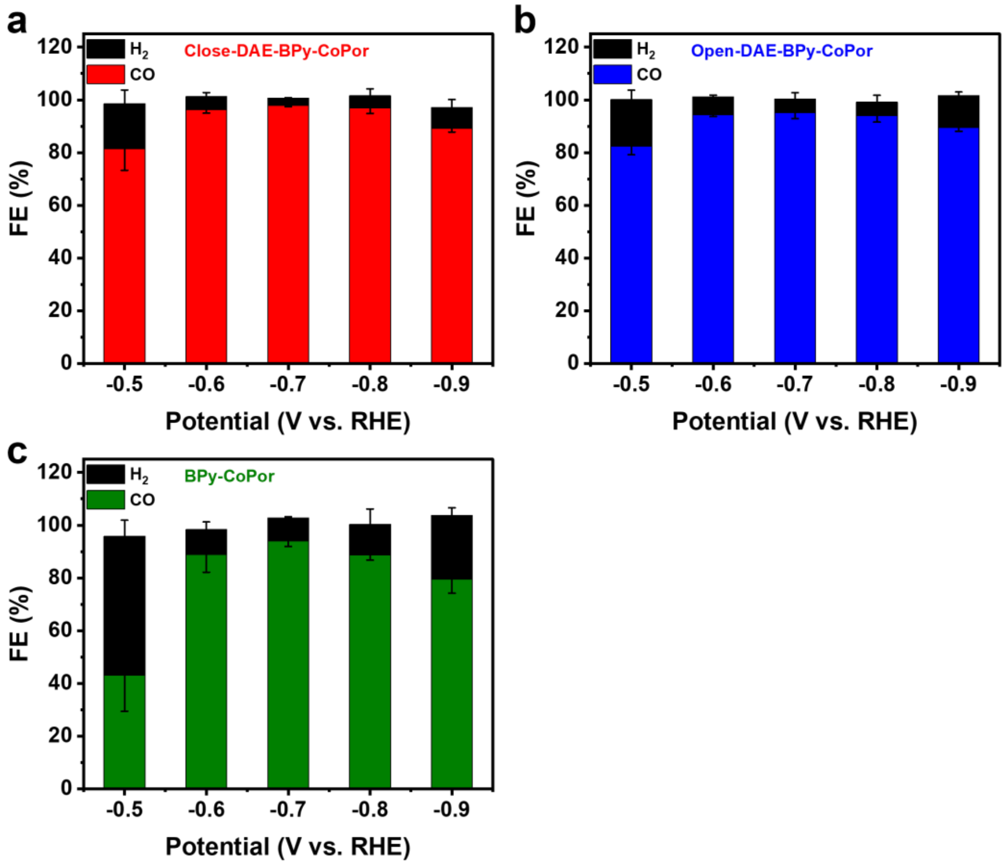

Supplementary Figure 25. The all Faradaic efficiencies of a close-DAE-BPy-CoPor, b open-DAE-BPy-CoPor and c BPy-CoPor under pure CO_2.

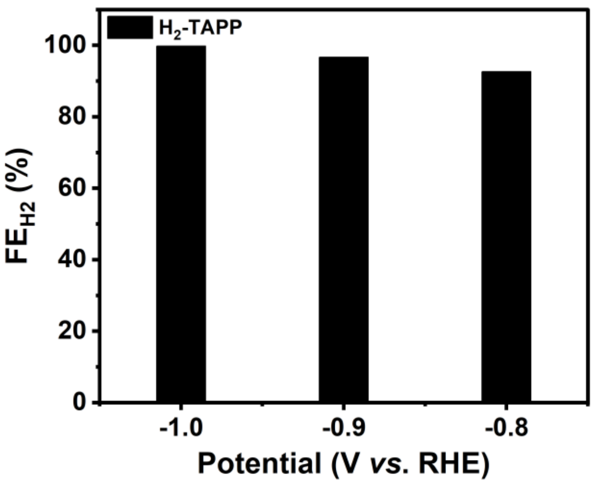

Supplementary Figure 26. The CO_2RR performance of H_2-TAPP in the CO_2-saturated 0.5 M $KHCO_3$.

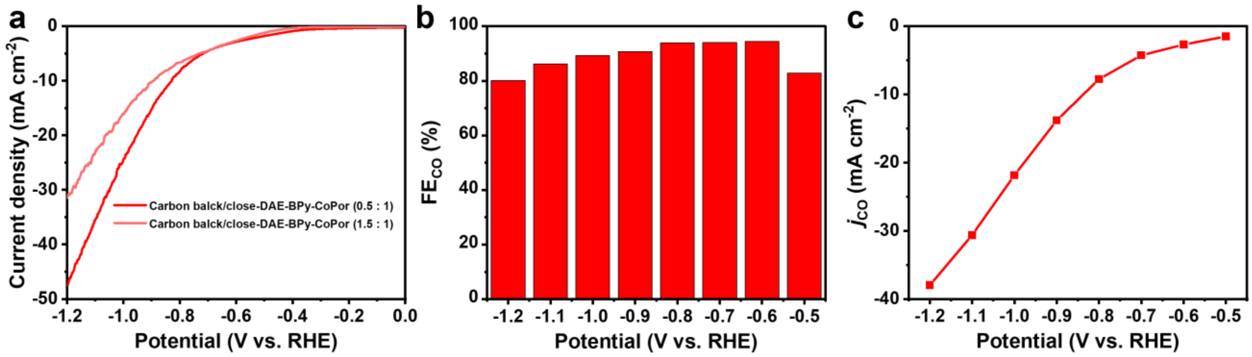

Supplementary Figure 27. a Linear sweep voltammetry curves of carbon black/close-DAE-BPy-CoPor (0.5 : 1) and carbon black/close-DAE-BPy-CoPor (1.5 : 1), **b** the CO Faradic efficiency and **c** the CO partial current density of carbon black/close-DAE-BPy-CoPor (1.5 : 1).

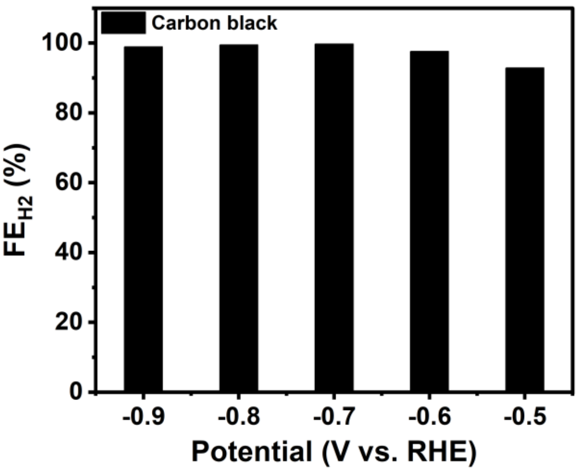

Supplementary Figure 28. The CO_2RR performance of carbon black in the CO_2-saturated 0.5 M $KHCO_3$.

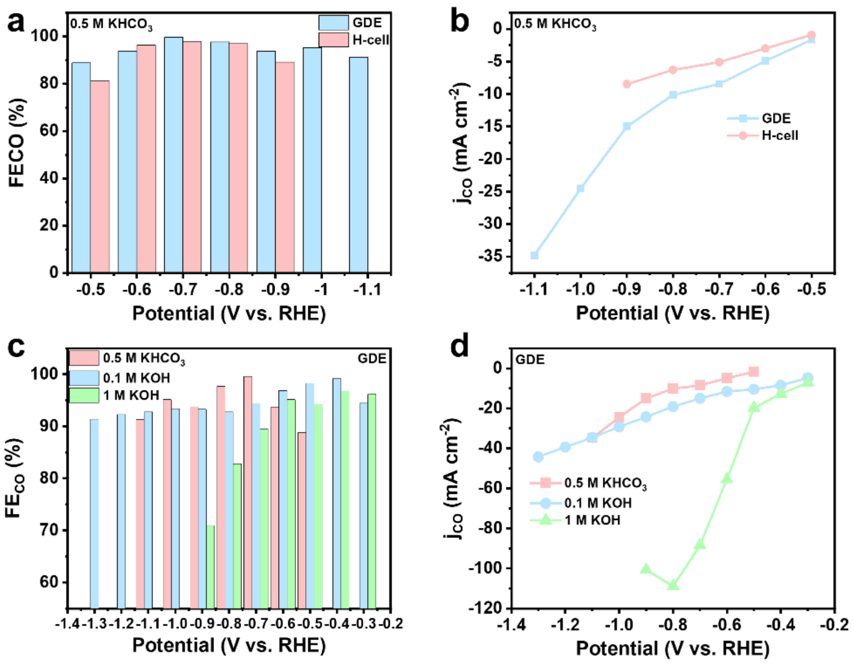

Supplementary Figure 29. The electrocatalytic CO_2RR performances of close-DAE-BPy-CoPor. **a** The FE_{CO} and **b** the j_{CO} in 0.5 M $KHCO_3$ using GDE and H-cell. **c** The FE_{CO} and **d** the j_{CO} in 0.5 M $KHCO_3$, 0.1 M KOH and 1 M KOH using GDE.

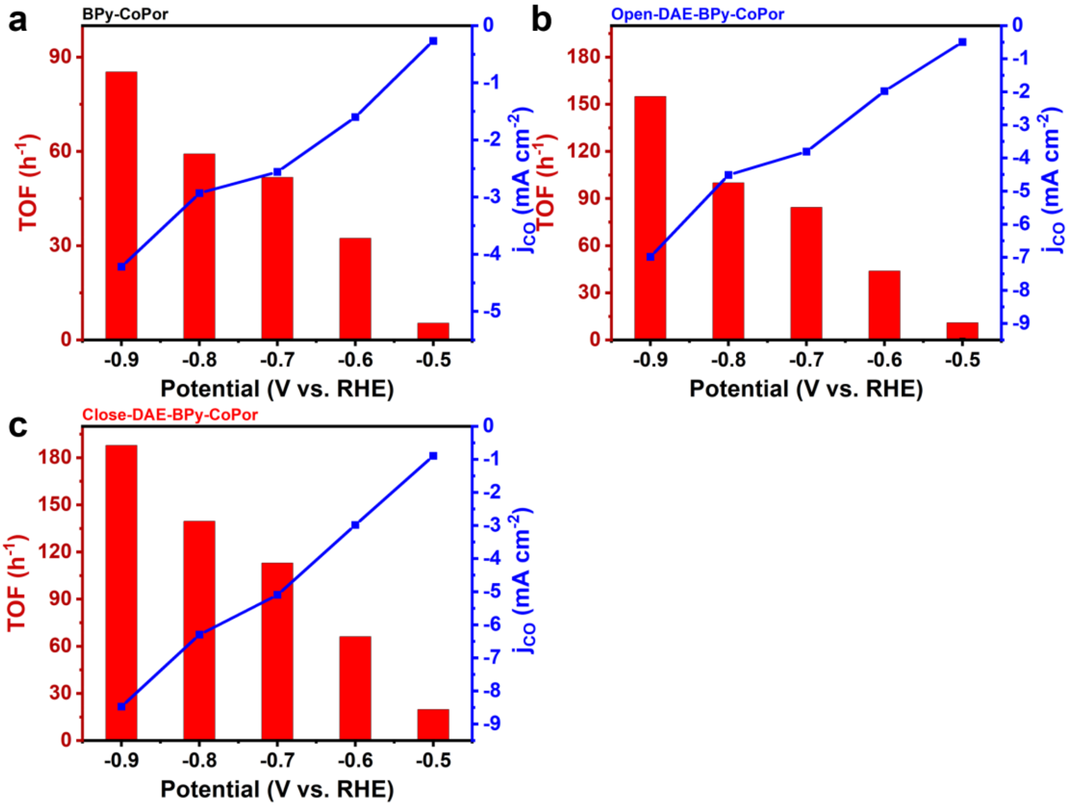

Supplementary Figure 30. The turnover frequency (TOF) of **a** BPy-CoPor, **b** open-DAE-BPy-CoPor and **c** close-DAE-BPy-CoPor at various applied potential.

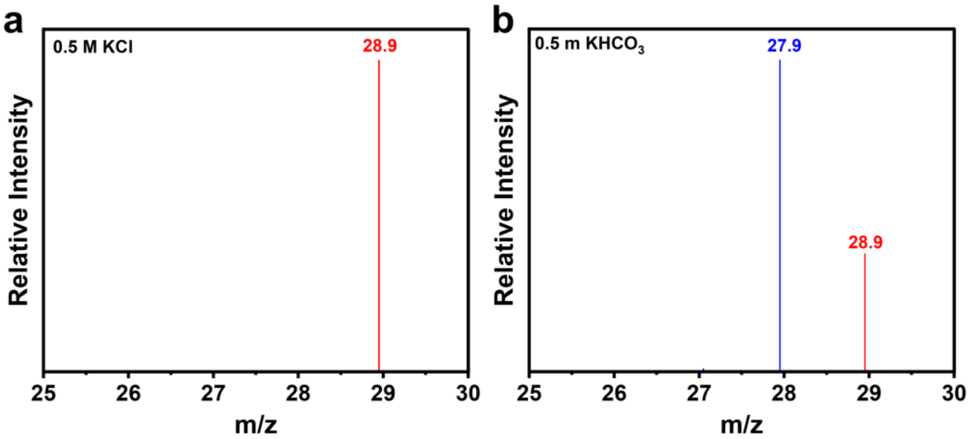

Supplementary Figure 31. Mass spectra of CO in $^{13}CO_2$-saturated **a** 0.5 M KCl and **b** 0.5 M $KHCO_3$ for close-DAE-BPy-CoPor.

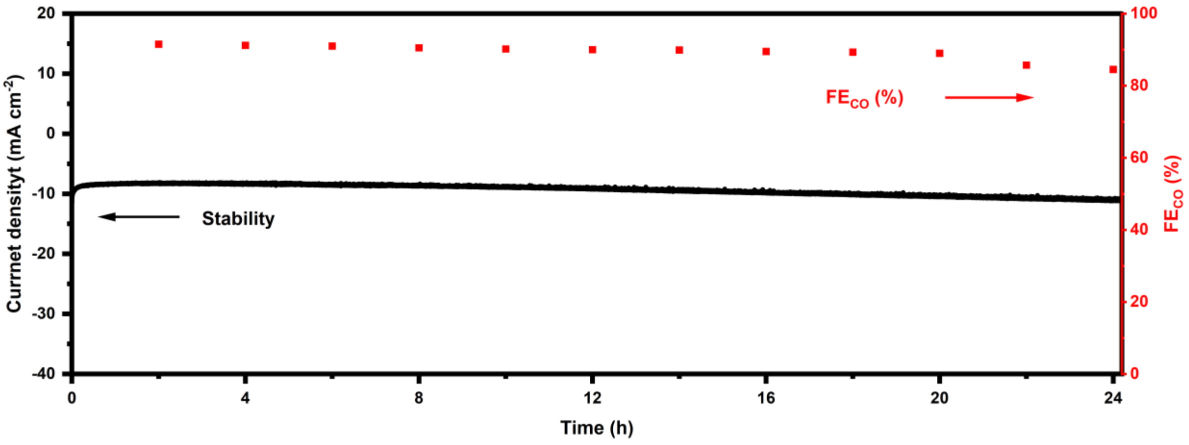

Supplementary Figure 32. Stability of close-DAE-BPy-CoPor at -0.9 V vs. RHE under CO_2-saturated 0.5 M $KHCO_3$ aqueous solution.

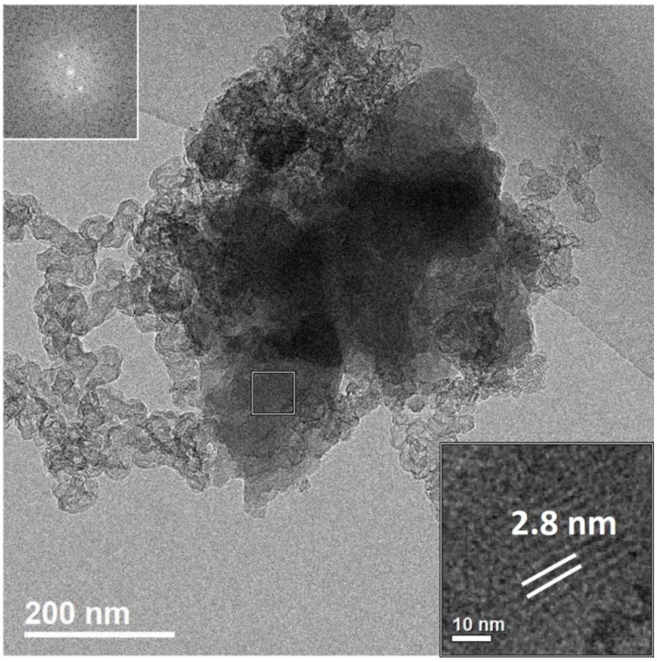

Supplementary Figure 33. The HRTEM image of close-DAE-BPy-CoPor after long-term stability test.

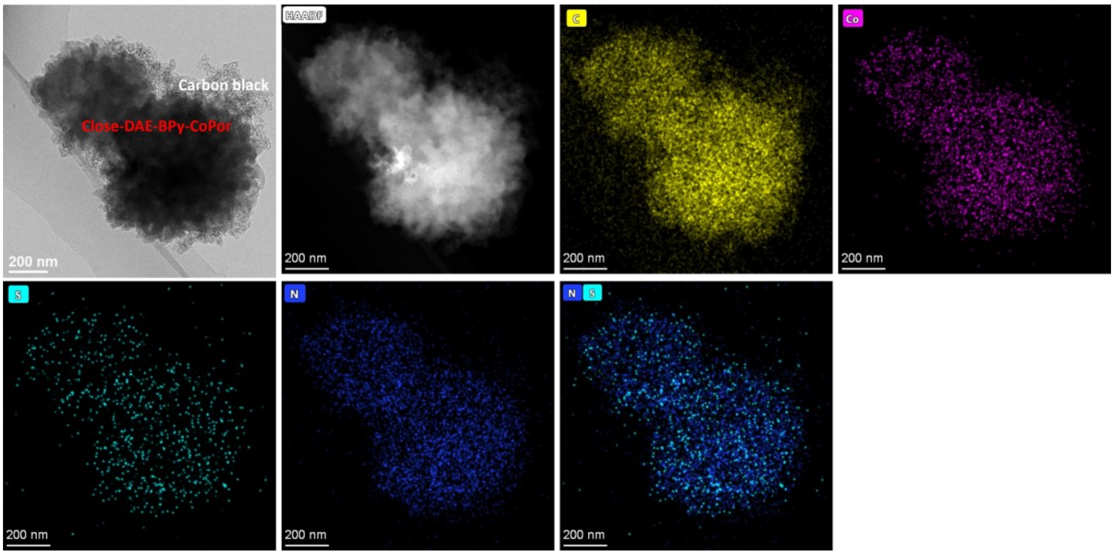

Supplementary Figure 34. The TEM image and EDX elemental mapping of close-DAE-BPy-CoPor after long-term stability test.

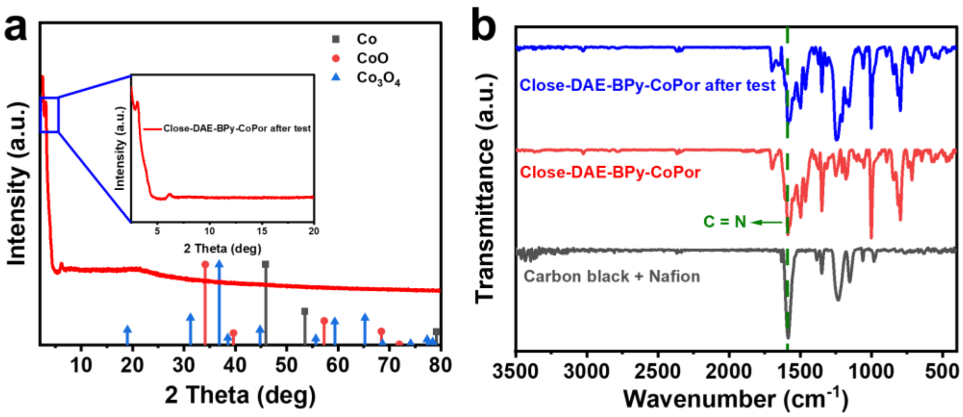

Supplementary Figure 35. a The PXRD pattern close-DAE-BPy-CoPor after CO_2RR. **b** The FT-IR of close-DAE-BPy-CoPor, close-DAE-BPy-CoPor after CO_2RR and carbon black mixed with Nafion.

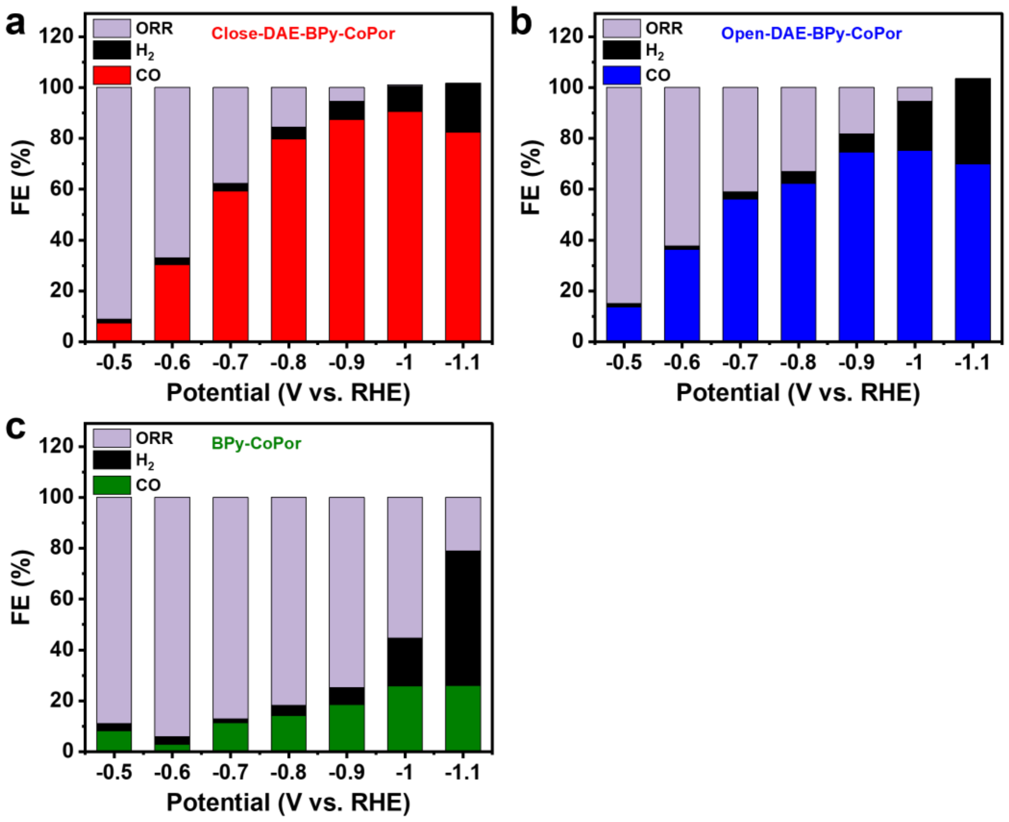

Supplementary Figure 36. The all Faradaic efficiencies of **a** close-DAE-BPy-CoPor, **b** open-DAE-BPy-CoPor and **c** BPy-CoPor under aerobic conditions. Due to the product of ORR is hard to detect in the aqueous solution, the FE of ORR is additional part of the FE of CO and H_2 (FE_{ORR} = 100% - (FE_{CO} + FE_{H2})).

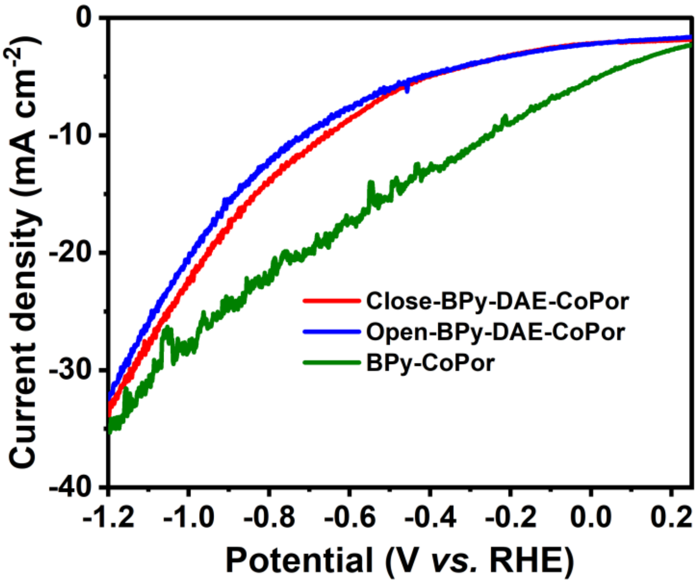

Supplementary Figure 37. The LSV of close-DAE-BPy-CoPor, open-DAE-BPy-CoPor and BPy-CoPor in the CO_2RR with co-fedding CO_2 and O_2.

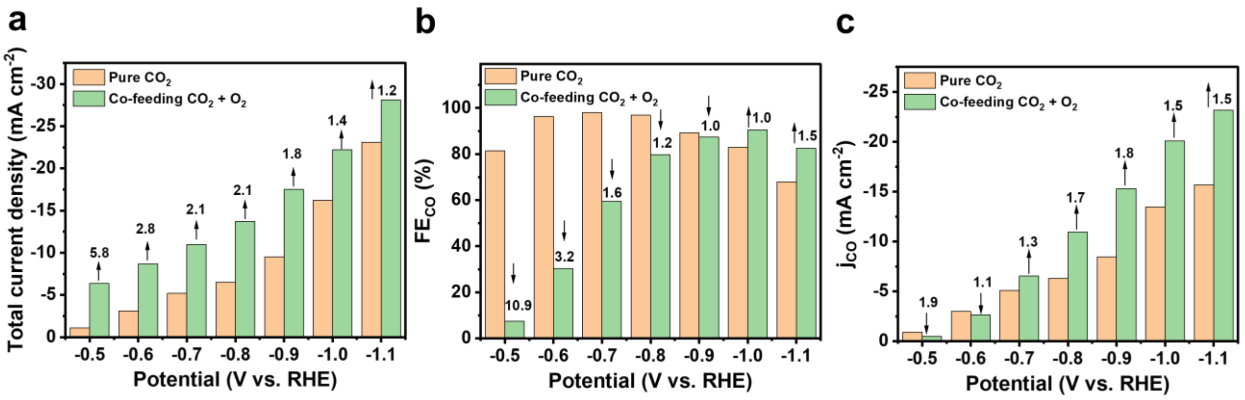

Supplementary Figure 38. The CO_2RR performance of close-DAE-BPy-CoPor under pure CO_2 and co-feeding $CO_2 + O_2$ condition. **a** total current density. **b** FE_{CO}. **c** j_{CO}. The numbers stand for the enhancement relative to the rates at pure CO_2.

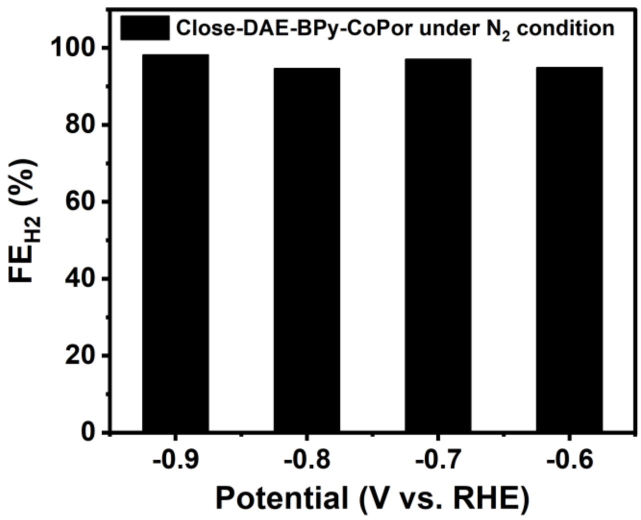

Supplementary Figure 39. The CO_2RR performance of close-DAE-BPy-CoPor under pure N_2 feeding environment.

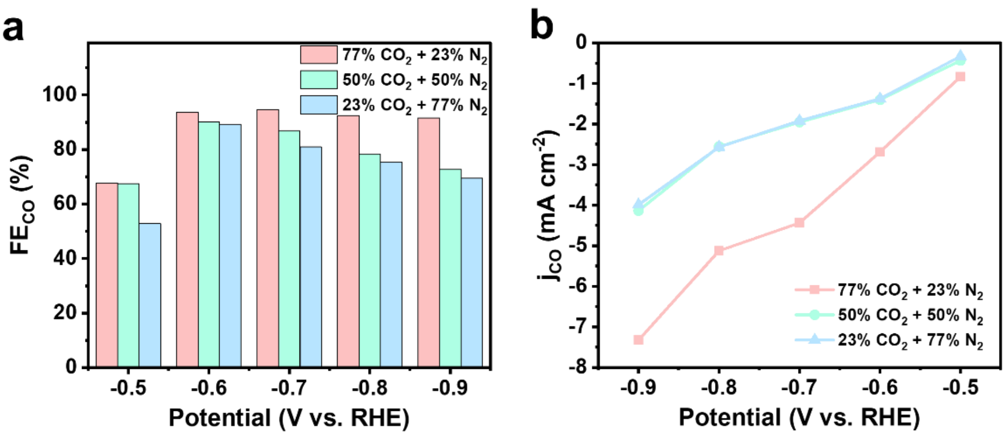

Supplementary Figure 40. The FE_{CO} **a** and j_{CO} **b** of close-DAE-BPy-CoPor under 77% CO_2 + 23% N_2, 50% CO_2 + 50% N_2 and 23% CO_2 + 77% N_2 feeding environment.

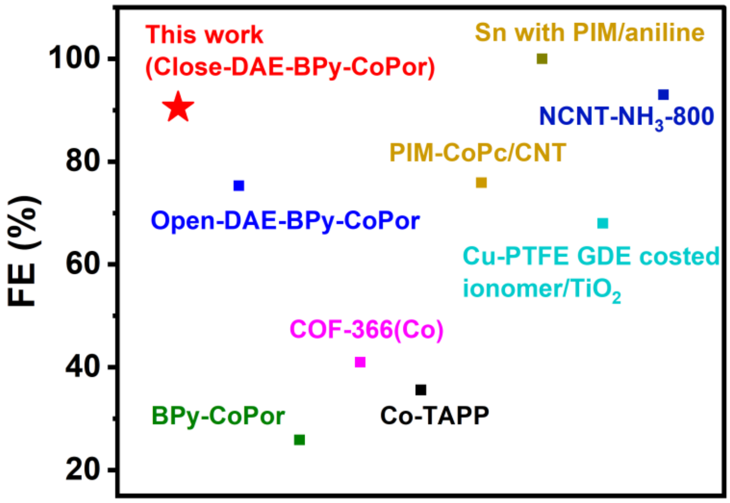

Supplementary Figure 41. The comparison of the optimal FE among the close-DAE-BPy-CoPor, open-DAE-BPy-CoPor, BPy-CoPor, Co-TAPP, COF-366(Co) and the reported electrocatalysts evaluated in the co-feeding CO_2 and O_2[1-4].

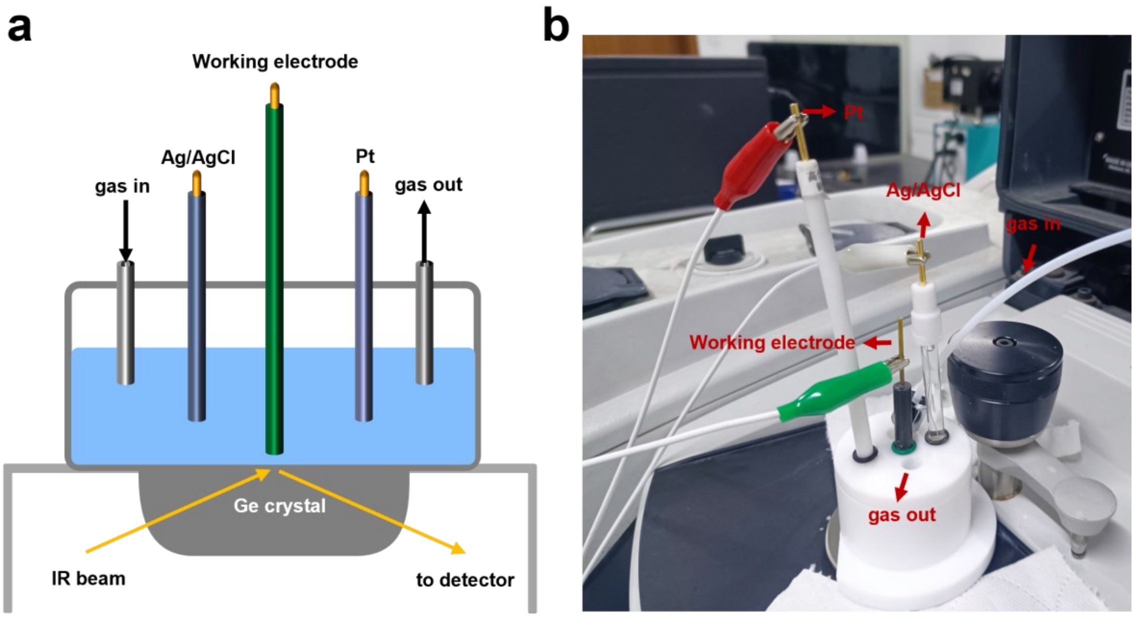

Supplementary Figure 42. a The schematic illustration home-made cell used for operando ATR-FTIR measurements. **b** Cell in use of the home-made cell.

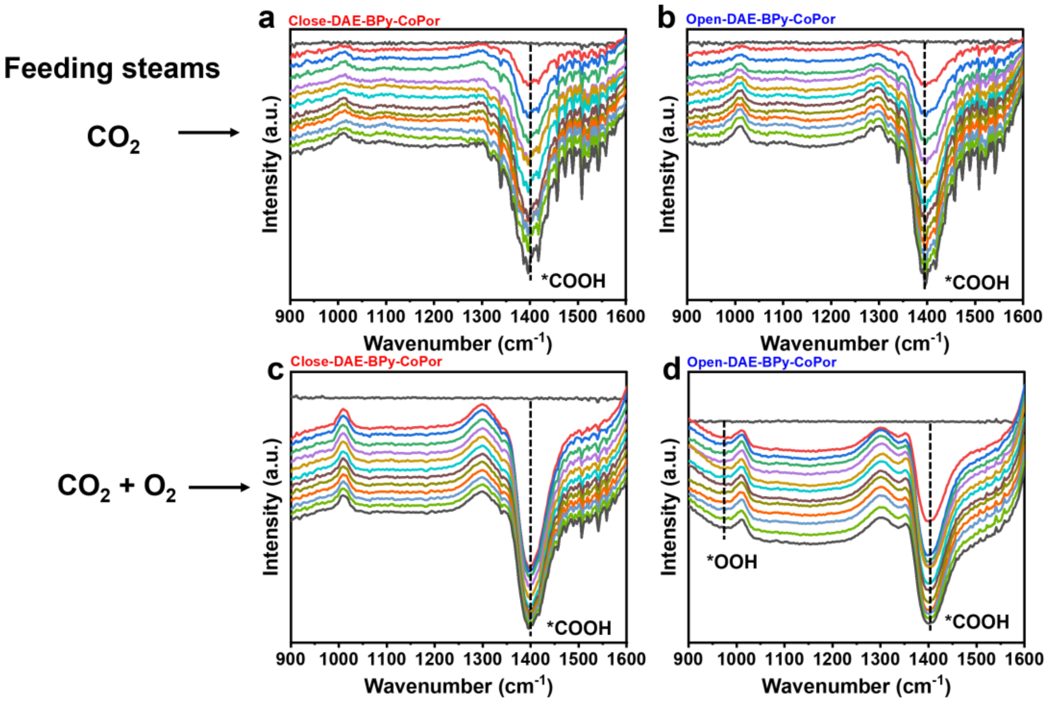

Supplementary Figure 43. Operando ATR-FTIR spectra on close-DAE-BPy-CoPor in the CO_2 saturated 0.5 M $KHCO_3$ **a** or co-feeding CO_2 and 5% O_2 **c**. Operando ATR-FTIR spectra on open-DAE-BPy-CoPor in the CO_2 saturated 0.5 M $KHCO_3$ **b** or co-feeding CO_2 and 5% O_2 **d**.

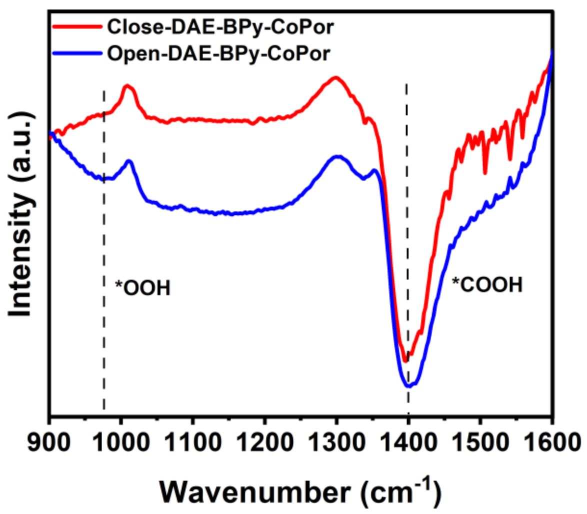

Supplementary Figure 44. Operando ATR-FTIR spectra on close-DAE-BPy-CoPor and in open-DAE-BPy-CoPor in the CO_2 saturated 0.5 M $KHCO_3$ and co-feeding CO_2 and 5% O_2.

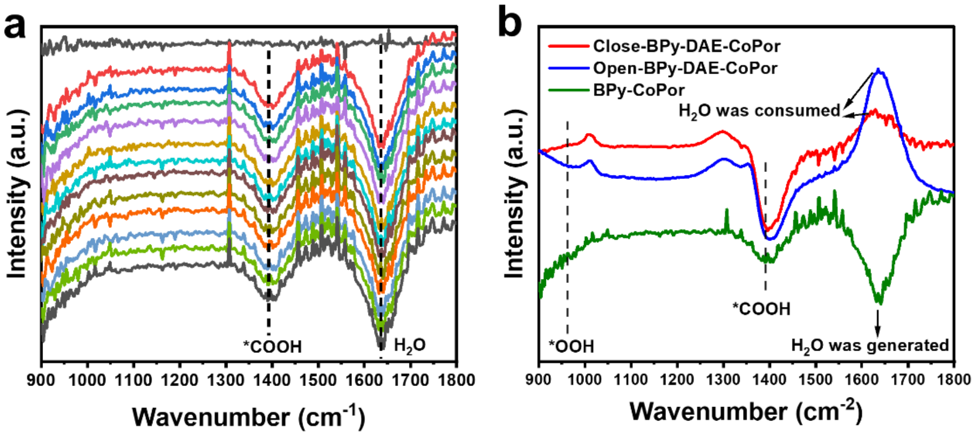

Supplementary Figure 45. a Operando ATR-FTIR spectra on BPy-CoPor in the co-feeding CO_2 and 5% O_2 0.5 M $KHCO_3$. **b** The comparison operando ATR-FTIR spectra of close-DAE-BPy-CoPor, open-DAE-BPy-CoPor and BPy-CoPor in the co-feeding CO_2 and 5% O_2 0.5 M $KHCO_3$.

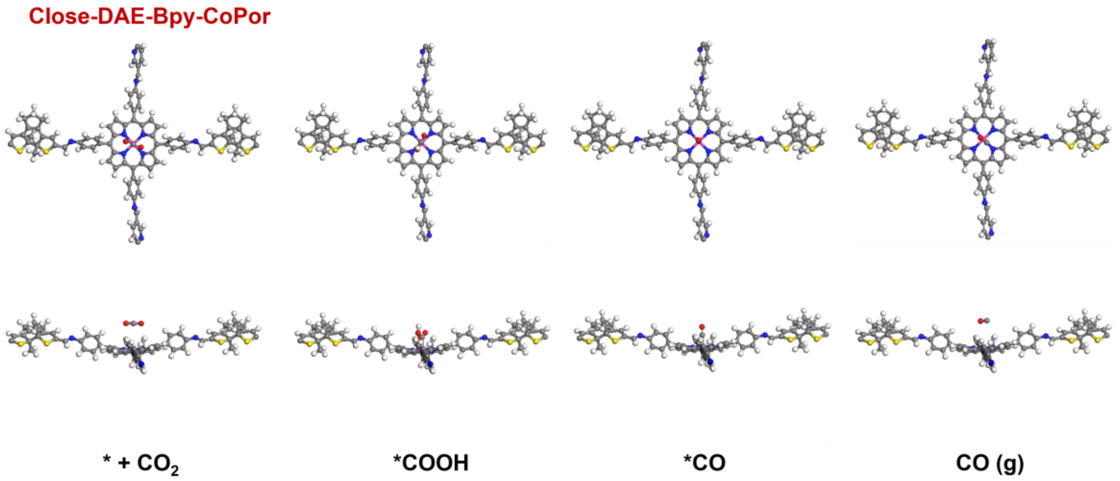

Supplementary Figure 46. The DFT calculated close-DAE-BPy-CoPor structures of various states for CO_2RR.

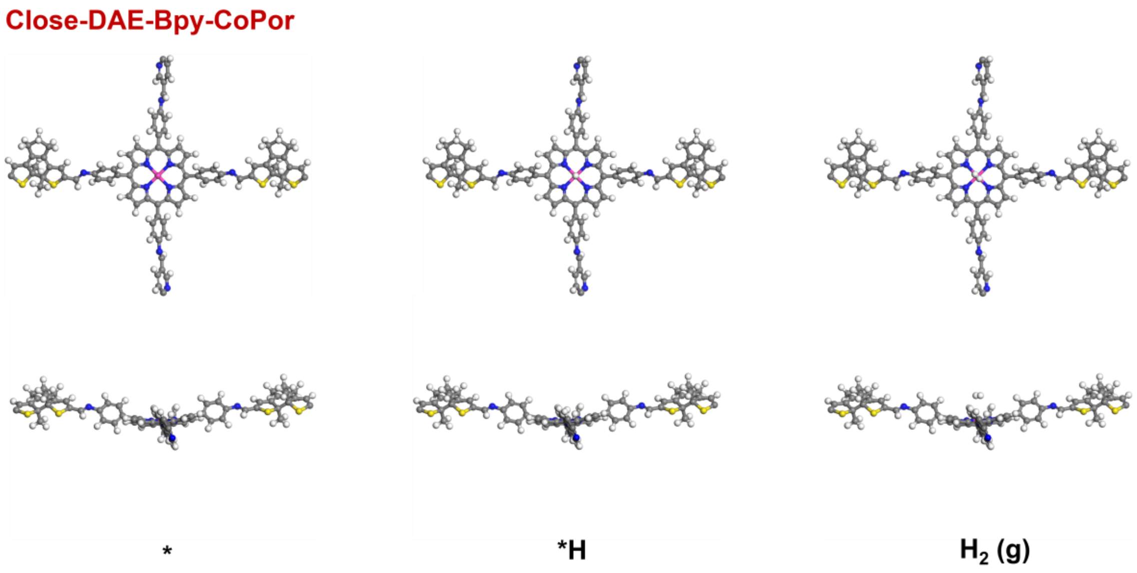

Supplementary Figure 47. The DFT calculated close-DAE-BPy-CoPor structures of various states for HER.

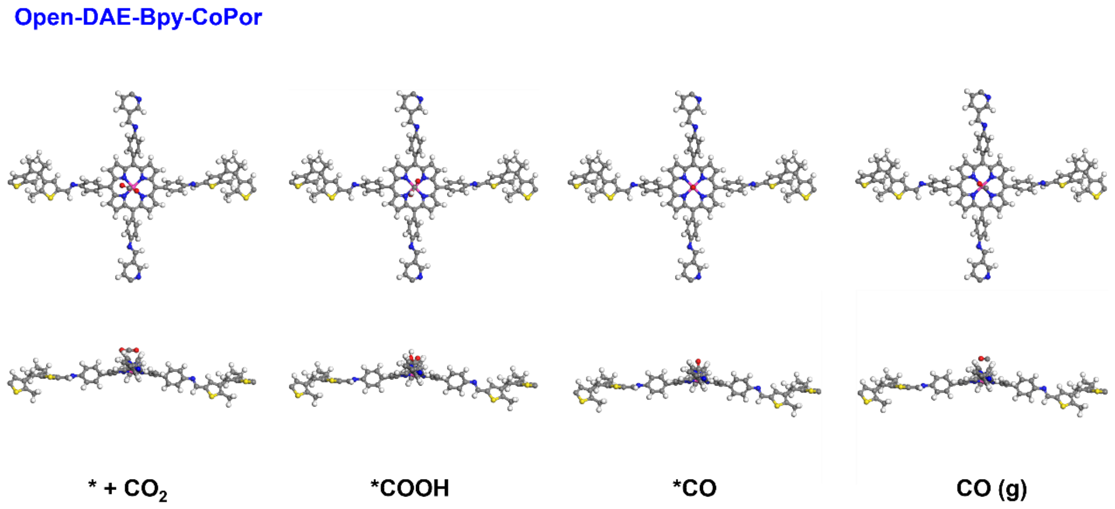

Supplementary Figure 48. The DFT calculated open-DAE-BPy-CoPor structures of various states for CO_2RR.

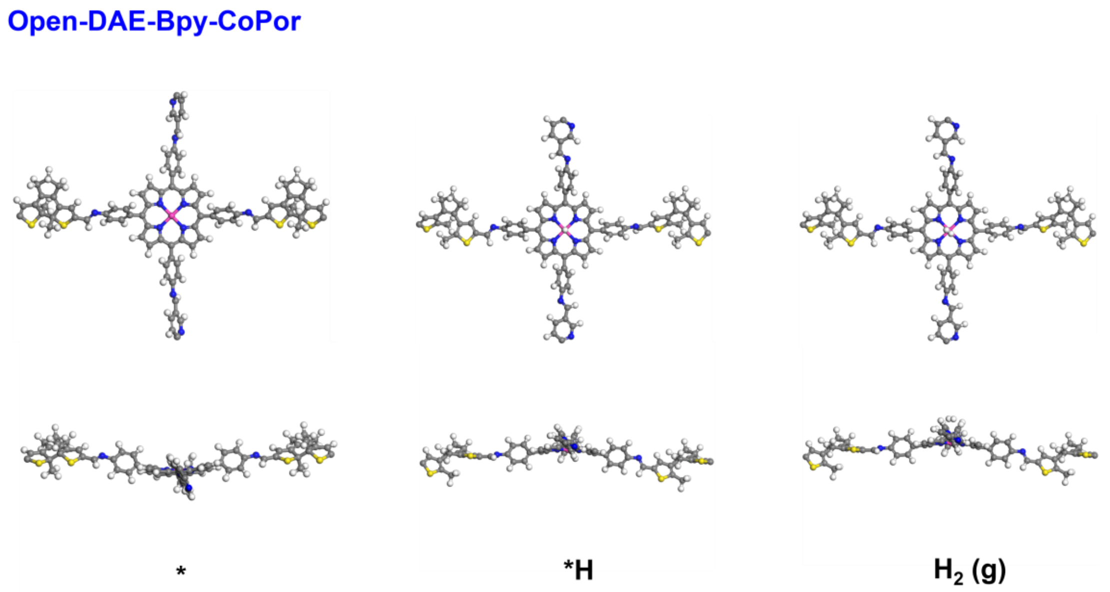

Supplementary Figure 49. The DFT calculated open-DAE-BPy-CoPor structures of various states for HER.

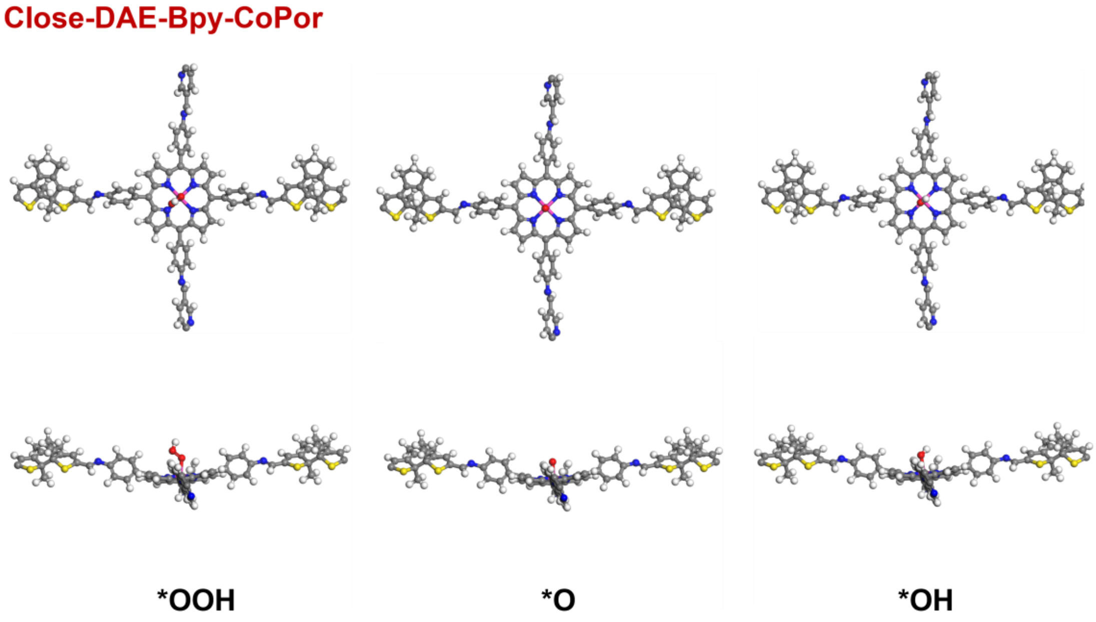

Supplementary Figure 50. The DFT calculated close-DAE-BPy-CoPor structures of various states for ORR.

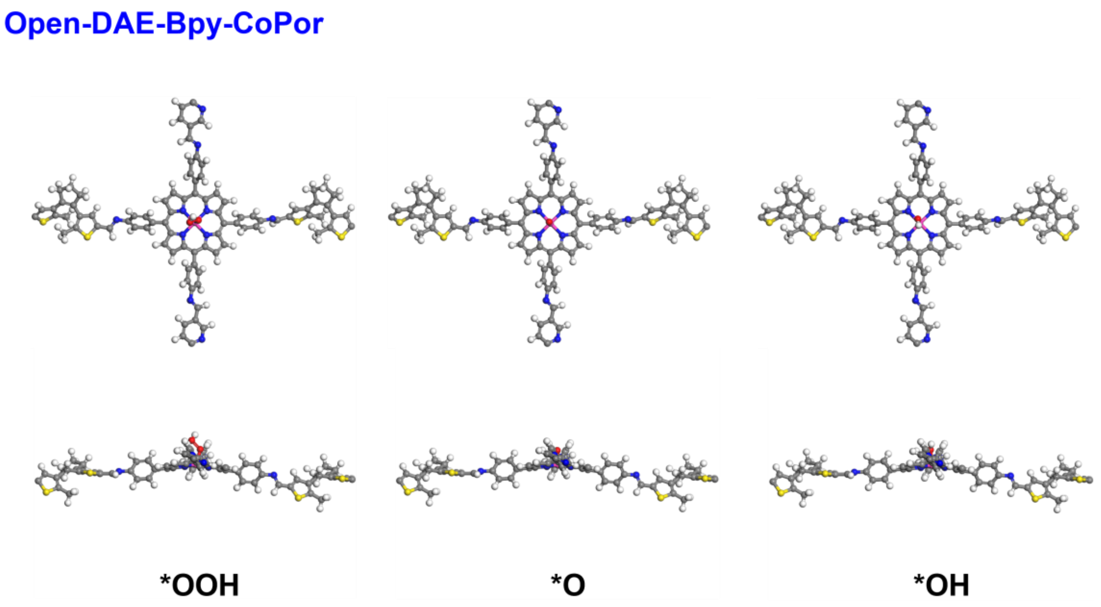

Supplementary Figure 51. The DFT calculated open-DAE-BPy-CoPor structures of various states for ORR.

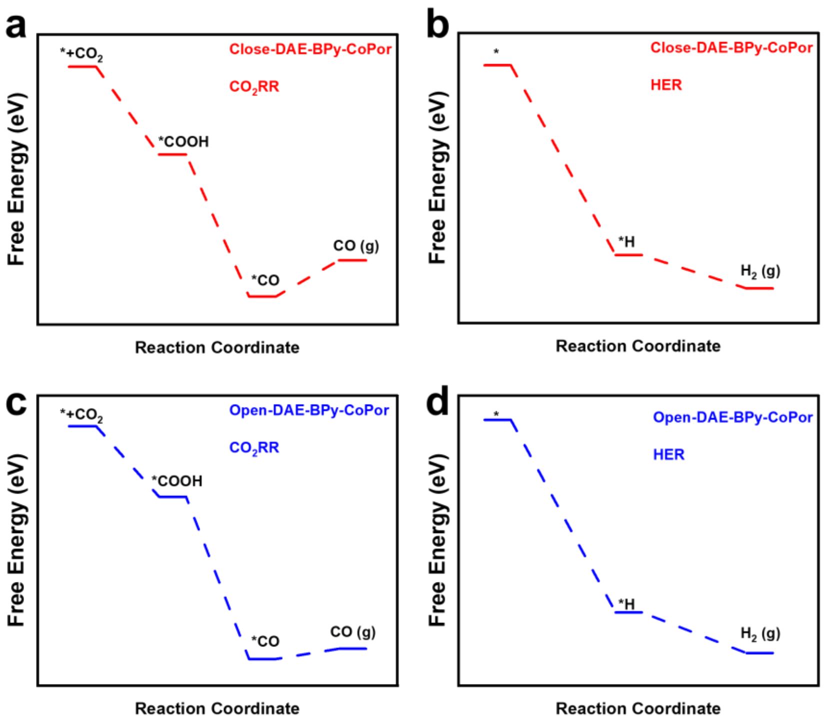

Supplementary Figure 52. Energy diagrams **a** CO_2RR and **b** HER of close-DAE-BPy-CoPor. **c** CO_2RR and **d** HER of open-DAE-BPy-CoPor vs the electrode potential at -0.7 V in H-cell.

The effect of a bias on all states involving an electron in the electrode was considered, by shifting the energy of this state by $\Delta G_U = -eU$, where U is the electrode potential.

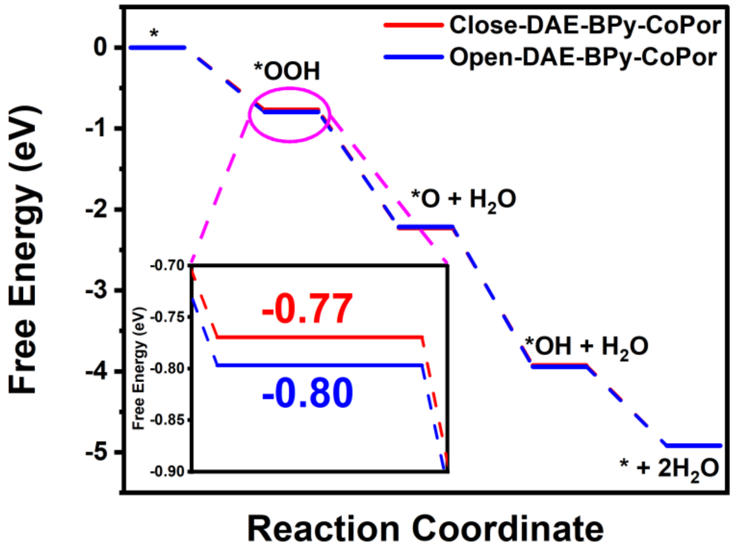

Supplementary Figure 53. The free energy of ORR pathways for close-DAE-BPy-CoPor and open-DAE-BPy-CoPor.

Supplementary Table 1. The structure model of open-DAE-BPy-CoPor.

**open-DAE-BPy-CoPor** Space group: *P1*<br>a = 29.6 Å, b = 29.8 Å, c = 4.58 Å<br>α = 81.4º, β = 73.6º, γ = 84.5º			
Atom	x	y	z
C1	0.25945	-0.74228	0.66886
C2	0.2908	-0.77562	0.64366
C3	0.33297	-0.75705	0.65536
N4	0.32645	-0.71195	0.67928
C5	0.28129	-0.70219	0.69894
C6	0.29089	-0.55088	0.77466
C7	0.25808	-0.58054	0.8125
C8	0.28055	-0.62191	0.74548
N9	0.32672	-0.61884	0.66676
C10	0.33263	-0.57441	0.67373
C11	0.25777	-0.66037	0.7461
C12	0.49124	-0.59231	0.34919
C13	0.46008	-0.55904	0.35624
C14	0.41561	-0.57924	0.48241
N15	0.42099	-0.62523	0.52771
C16	0.46694	-0.63427	0.46185
C17	0.37448	-0.55375	0.57514
C18	0.45365	-0.78932	0.65975
C19	0.48579	-0.75931	0.63282
C20	0.46543	-0.71641	0.58097
N21	0.4206	-0.71881	0.59069
C22	0.41402	-0.764	0.63452
C23	0.37332	-0.78343	0.65178
C24	0.48895	-0.67661	0.5166
C25	0.37578	-0.50345	0.56922
C26	0.53804	-0.6779	0.52149
C27	0.37282	-0.83375	0.66411
C28	0.20764	-0.65563	0.76903
C29	0.40397	-0.48624	0.71741
C30	0.40738	-0.43947	0.69895
C31	0.38056	-0.40887	0.54924
C32	0.35162	-0.4257	0.40494
C33	0.34994	-0.47256	0.4103
C34	0.57301	-0.7046	0.34225
C35	0.61874	-0.70309	0.34774
C36	0.63032	-0.67382	0.52354
C37	0.59524	-0.64723	0.70174
C38	0.54988	-0.65007	0.70423
C39	0.34316	-0.85947	0.91057
C40	0.34168	-0.90631	0.91394
C41	0.37024	-0.92834	0.67434
C42	0.40076	-0.90278	0.4312
C43	0.40166	-0.85595	0.42586
C44	0.19203	-0.62403	0.55696

C45	0.1461	-0.6201	0.55962
C46	0.11477	-0.64861	0.76858
C47	0.12949	-0.6797	0.98984
C48	0.17575	-0.68279	0.99158
N49	0.38492	-0.3611	0.53749
N50	0.67701	-0.67394	0.53643
N51	0.3668	-0.9762	0.68067
N52	0.06913	-0.64526	0.7362
C53	0.35241	-0.32987	0.52557
C54	0.6936	-0.64027	0.61301
C55	0.3927	-0.99957	0.47328
C56	0.0368	-0.67322	0.85574
C57	0.3608	-0.28158	0.51163
C58	0.40548	-0.26636	0.42298
C59	0.41237	-0.22041	0.4097
C60	0.37433	-0.18964	0.48854
N61	0.33131	-0.20526	0.57753
C62	0.32374	-0.24977	0.58799
C63	0.37998	-0.14083	0.48187
C64	0.41526	-0.11803	0.25868
C65	0.419	-0.07175	0.25845
C66	0.38802	-0.04837	0.4803
C67	0.35305	-0.0722	0.69895
N68	0.34978	-0.11723	0.69309
Co69	0.37367	-0.66874	0.61673
C70	0.73881	-0.6442	0.67029
C71	0.77062	-0.68007	0.61061
C72	0.80972	-0.67606	0.70304
C73	0.80383	-0.63667	0.84337
S74	0.75441	-0.60427	0.84251
C75	0.84819	-0.7128	0.66632
C76	0.83602	-0.75814	0.61509
C77	0.87682	-0.79095	0.62384
C78	0.91308	-0.76278	0.66228
C79	0.89285	-0.71478	0.67542
C80	0.9252	-0.68022	0.69324
C81	0.95959	-0.69379	0.83841
C82	0.99429	-0.66427	0.76064
S83	0.98649	-0.61778	0.51304
C84	0.93606	-0.63826	0.51184
C85	0.90854	-0.60945	0.32989
C86	0.8339	-0.62319	1.01118
H87	0.22481	-0.74487	0.6566
H88	0.2857	-0.80964	0.60921
H89	0.28424	-0.51575	0.81487
H90	0.22152	-0.57227	0.88342
H91	0.52785	-0.5885	0.26399
H92	0.46716	-0.52373	0.27784
H93	0.45893	-0.82587	0.69951
H94	0.52019	-0.76864	0.65125

H95	0.42386	-0.50912	0.84464
H96	0.43005	-0.42698	0.80945
H97	0.33169	-0.40279	0.27824
H98	0.32874	-0.48473	0.2877
H99	0.5651	-0.72594	0.19434
H100	0.64525	-0.72409	0.21075
H101	0.60209	-0.62569	0.85181
H102	0.52362	-0.62963	0.84485
H103	0.32094	-0.84315	1.09887
H104	0.31824	-0.92553	1.10381
H105	0.42328	-0.91871	0.24222
H106	0.42487	-0.83684	0.23561
H107	0.21572	-0.60224	0.38967
H108	0.1351	-0.59582	0.38971
H109	0.10576	-0.7014	1.16005
H110	0.18685	-0.70678	1.16239
H111	0.31814	-0.33905	0.54057
H112	0.67339	-0.60879	0.65311
H113	0.04182	-0.70404	1.00342
H114	0.43503	-0.28986	0.36232
H115	0.44706	-0.2093	0.34393
H116	0.28883	-0.26038	0.65888
H117	0.43906	-0.13546	0.08227
H118	0.44597	-0.05413	0.08422
H119	0.32831	-0.05516	0.87308
H120	0.765	-0.70783	0.50069
H121	0.82931	-0.75552	0.38659
H122	0.80527	-0.77009	0.79827
H123	0.88919	-0.80679	0.40845
H124	0.86747	-0.8183	0.82067
H125	0.94448	-0.76425	0.46802
H126	0.92187	-0.77661	0.87855
H127	0.96132	-0.72561	0.98253
H128	0.87382	-0.61995	0.40606
H129	0.92342	-0.61135	0.08393
H130	0.90747	-0.57349	0.36981
H131	0.85624	-0.59626	0.87288
H132	0.85332	-0.65368	1.08761
H133	0.81251	-0.60774	1.21276

Supplementary Table 2. ICP-MS analysis result of BPy-CoPor and open-DAE-BPy-CoPor.

COFs		Co (wt.%)
BPy-CoPor	Calculated	5.44
	Experimental	5.36
Open-DAE-BPy-CoPor	Calculated	4.97
	Experimental	4.74

Supplementary Table 3. Fitting results from EXAFS analysis of open-DAE-BPy-CoPor. (CN: coordination number; R: distance between absorber and backscatter atoms; σ^2: Debye-Waller factor (a measure of thermal and static disorder in absorber-scatterer distances); $\triangle E_0$: the inner potential correction; R factor is used to value the goodness of the fitting.)

Sample	Path	CN	R(Å)	$\sigma^2(10^{-3}$ Å$^2)$	$\triangle E_0$ (eV)	R factor
Open-DAE-BPy-CoPor	Co-N	4.3 ± 0.3	1.95	6.32	-6.48	0.02
Close-DAE-Bpy-CoPor	Co-N	4.0 ± 0.6	1.93	4.97	-1.21	0.003

Supplementary Table 4. The summary of CO_2 electroreduction performances for reported electrocatalysts and this work.

Catalyst	electrolyte	Highest FE_{CO} (%)	j_{CO} (mA cm^{-2})	Stability (h)	Ref.
Close-DAE-BPy-CoPor	0.5 M $KHCO_3$	98.0	-8.47 (-0.9 V)	24	This work
Carbon black/Close-DAE-BPy-CoPor (1.5 : 1)	0.5 M $KHCO_3$	80.2	-38.0 (-1.2 V)	NA	This work
Open-DAE-BPy-CoPor	0.5 M $KHCO_3$	95.2	-6.99 (-0.9 V)	NA	This work
BPy-CoPor	0.5 M $KHCO_3$	94.1	-5.30 (-0.9 V)	NA	This work
ViB_{12}@rGO	0.5 M $KHCO_3$	94.5	-6.24 (-0.8 V)	10	*ACS Appl Mater Interfaces* 12, 41288-41293 (2020)
Co-TTCOF	0.5 M $KHCO_3$	91.3	-1.84 (-0.7 V)	40	*Nat Commun 11, 497 (2020)*
COF-366-Co	0.5 M $KHCO_3$	90.0	-1.8 (-1.1 V)	24	*Science 349, 1208 (2015)*
COF-367-Co	0.5 M $KHCO_3$	91.0	-3.3 (-1.1 V)	24	*Science 349, 1208 (2015)*
TTF-Por(Co)-COF	0.5 M $KHCO_3$	70.0	-6.88 (-0.9 V)	10	*ACS Energy Letters* ***5**, 1005-1012 (2020)*
Co-TPP-cov	0.5 M $KHCO_3$	67.0	-1.065 (-0.63 V)	4	*Angew. Chem. Int. Ed. 56, 6468-6472 (2017)*
Co-TPP/CNT	0.5 M $KHCO_3$	91.0	-3.2 (-0.66 V)	12	*Angew. Chem. Int. Ed. 58, 6595-6599 (2019)*
Co-Bpy-COF-Ru1/2	0.5 M $KHCO_3$	96.7	about -9 (-0.7 V)	13	*J Am Chem Soc*, (2023)
$Ni-N_3-V$	0.5 M $KHCO_3$	94.0	-48 (-0.8 V)	14	*Angew. Chem., Int. Ed.* **2019,** 59, 1961-1965
$C-Zn_1-Ni_4$ ZIF-8	0.5 M $KHCO_3$	98.0	-55 (-0.8 V)	12	*Energy Environ. Sci.* **2018**, 11, 1204-1210
rGO-PEI-MoSx	0.5 M $NaHCO_3$	85.1	-55 (-0.65 V)	3	Energy Environ. Sci. **2016**, 9, 216
CoPcPDQ-COF	0.5 M $KHCO_3$	96.0	-49.4 (-0.66 V)	24	Angew. Chem. Int. Ed. **2020**, 59, 16587-16593

Supplementary Note 1: Calculation methods

To confirm the speculation of oxygen passivation strategy, the mechanism of CO_2RR, HER and ORR on the open-DAE-BPy-CoPor and closed-DAE-BPy-CoPor were calculated by density functional theory (DFT) calculations through Vienna Ab initio Simulation Package (VASP)[5-7]. Pseudopotentials were conducted by the Perdew-Burke-Ernzerh (PBE) exchange-correlation functional[8] and the projector-augmented wave (PAW)[9]. In the intermediates optimizations, the convergence criteria and the cutoff energy of plane wave basis were set to 1×10^{-4} eV and 400 eV, respectively. The Monkhorst–Pack k-mesh of 2×2×1 was adopted, and the Van der Waals (vdW) correction was adopted by Grimme (DFT+D3)[10].

The open-DAE-BPy-CoPor and closed-DAE-BPy-CoPor were modeled using two-layer slab, respectively. The top layer was fully relaxed, and the remaining layer was fixed in optimization. To avoid the periodic interactions of the system, a vacuum region of 25 Å between two repeated slabs was used in the direction perpendicular to the surface.

The free energy change (ΔG) of each electrochemical step was calculated based on the computational hydrogen electrode (CHE) model. A correction of −0.51 and 0.13 eV for CO and CO_2 was performed due to the defect of the PBE functional in the C=O bond description. The solvation effects were considered to stabilize at 0.25 and 0.10 eV for *COOH and *CO, respectively.[11] The Gibbs free energies of intermediates were calculated by the equation $\Delta G = \Delta G_0 + \Delta G(\text{pH})$. The ΔG_0 was calculated at 298.15 K by the VASPKIT package,[12] which according to $\Delta G_0 = \Delta E_{\text{DFT}} + \Delta E_{\text{ZPE}} - T\Delta S$. The E_{DFT}, E_{ZPE}, and S indicates the electronic energy, zero-point energy and entropy, respectively. The free energy of H^+ ions was corrected by the concentration dependence of the entropy: $\Delta G(\text{pH}) = -\text{kTln}[H^+] = \text{k}T\text{ln}10\times\text{pH}$.The experiment pH was 6.8 in this study.

Supplementary references

1 Cheng, Y., Hou, J. & Kang, P. Integrated Capture and Electroreduction of Flue Gas CO_2 to Formate Using Amine Functionalized SnOx Nanoparticles. *ACS Energy Lett.* **6**, 3352-3358 (2021).

2 Shi, H., Pan, H., Cheng, Y., Lu, S. & Kang, P. Imine-Nitrogen-Doped Carbon Nanotubes for the Electrocatalytic Reduction of Flue Gas CO_2. *ChemElectroChem* **8**, 1792-1797 (2021).

3 Xu, Y. *et al.* Oxygen-tolerant electroproduction of C2 products from simulated flue gas. *Energy Environ. Sci.* **13**, 554-561 (2020).

4 Lu, X. *et al.* A bio-inspired O_2-tolerant catalytic CO_2 reduction electrode. *Sci. Bull.* **64**, 1890-1895 (2019).

5 Kresse, G. & Hafner, J. Ab initio molecular dynamics for liquid metals. *Physical review B* **47**, 558 (1993).

6 Kresse, G. & Hafner, J. Ab initio molecular-dynamics simulation of the liquid-metal–amorphous-semiconductor transition in germanium. *Physical Review B* **49**, 14251 (1994).

7 Kresse, G. & Furthmüller, J. Efficient iterative schemes for ab initio total-energy calculations using a plane-wave basis set. *Physical review B* **54**, 11169 (1996).

8 Perdew, J. P., Burke, K. & Ernzerhof, M. Generalized gradient approximation made simple. *Phys. Rev. Lett.* **77**, 3865 (1996).

9 Blöchl, P. E. Projector augmented-wave method. *Physical review B* **50**, 17953 (1994).

10 Grimme, S. Density functional theory with London dispersion corrections. *Wiley Interdisciplinary Reviews: Computational Molecular Science* **1**, 211-228 (2011).

11 Cheng, H. *et al.* Atomically dispersed Ni/Cu dual sites for boosting the CO_2 reduction reaction. *ACS Catal.* **11**, 12673-12681 (2021).

12 Wang, V., Xu, N., Liu, J.-C., Tang, G. & Geng, W.-T. VASPKIT: A user-friendly interface facilitating high-throughput computing and analysis using VASP code. *Comput. Phys. Commun.* **267**, 108033 (2021).
