# Supplementary material for: Oxygen-tolerant CO2 electroreduction over covalent organic frameworks via photoswitching control oxygen passivation strategy
Source: Nat Commun. 2024 Feb 17;15:1479. doi: 10.1038/s41467-024-45959-9 (PMC10874412; doi:10.1038/s41467-024-45959-9)
Supplement: Supplementary file 3 — Description of Additional Supplementary Files [file 41467_2024_45959_MOESM3_ESM.pdf]

Description of Additional Supplementary Files

Supplementary Data 1: Coordinates for model slab of close-DAE-BPy-CoPor (Space group P1).
Supplementary Data 2: Coordinates for model slab of close-DAE-BPy-CoPor + CO_2 (Space group P1).
Supplementary Data 3: Coordinates for model slab of close-DAE-BPy-CoPor + *COOH (Space group P1).
Supplementary Data 4: Coordinates for model slab of close-DAE-BPy-CoPor - CO (Space group P1).
Supplementary Data 5: Coordinates for model slab of close-DAE-BPy-CoPor + CO (Space group P1).
Supplementary Data 6: Coordinates for model slab of close-DAE-BPy-CoPor - *H (Space group P1).
Supplementary Data 7: Coordinates for model slab of close-DAE-BPy-CoPor + H_2 (Space group P1).
Supplementary Data 8: Coordinates for model slab of close-DAE-BPy-CoPor - O_2 (Space group P1).
Supplementary Data 9: Coordinates for model slab of close-DAE-BPy-CoPor - *OOH (Space group P1).
Supplementary Data 10: Coordinates for model slab of close-DAE-BPy-CoPor - *O + H_2O (Space group P1).
Supplementary Data 11: Coordinates for model slab of close-DAE-BPy-CoPor - *OH + H_2O (Space group P1).
Supplementary Data 12: Coordinates for model slab of open-DAE-BPy-CoPor (Space group P1).
Supplementary Data 13: Coordinates for model slab of open-DAE-BPy-CoPor + CO_2 (Space group P1).
Supplementary Data 14: Coordinates for model slab of open-DAE-BPy-CoPor - *COOH (Space group P1).
Supplementary Data 15: Coordinates for model slab of open-DAE-BPy-CoPor - CO (Space group P1).
Supplementary Data 16: Coordinates for model slab of open-DAE-BPy-CoPor + CO (Space group P1).
Supplementary Data 17: Coordinates for model slab of open-DAE-BPy-CoPor - *H (Space group P1).

Supplementary Data 18: Coordinates for model slab of open-DAE-BPy-CoPor + H_2 (Space group P1).

Supplementary Data 19: Coordinates for model slab of open-DAE-BPy-CoPor - O_2 (Space group P1).

Supplementary Data 20: Coordinates for model slab of open-DAE-BPy-CoPor - *OOH (Space group P1).

Supplementary Data 21: Coordinates for model slab of open-DAE-BPy-CoPor - *O + H_2O (Space group P1).

Supplementary Data 22: Coordinates for model slab of open-DAE-BPy-CoPor - *OH + H_2O (Space group P1).
